# Initial SEI formation in LiBOB-, LiDFOB- and LiBF_4_-containing PEO electrolytes†

**DOI:** 10.1039/d3ta07175h

**Published:** 2024-03-19

**Authors:** Edvin K. W. Andersson, Liang-Ting Wu, Luca Bertoli, Yi-Chen Weng, Daniel Friesen, Kenza Elbouazzaoui, Sophia Bloch, Ruslan Ovsyannikov, Erika Giangrisostomi, Daniel Brandell, Jonas Mindemark, Jyh-Chiang Jiang, Maria Hahlin

**Affiliations:** a Department of Chemistry –Ångström Laboratory, Uppsala University Box 538 Uppsala 75121 Sweden maria.hahlin@kemi.uu.se; b Department of Chemical Engineering, National Taiwan University of Science and Technology Taipei 106 Taiwan; c Dipartimento di Chimica, Materiali e Ingegneria Chimica “Giulio Natta”, Politecnico di Milano Via Luigi Mancinelli 7 20131 Milan Italy; d Department of Physics and Astronomy, Uppsala University Box 516 Uppsala 75120 Sweden; e Institute for Methods and Instrumentation for Synchrotron Radiation Research, Helmholtz-Zentrum Berlin für Materialien und Energie Albert-Einstein-Str. 15 12489 Berlin Germany

## Abstract

A limiting factor for solid polymer electrolyte (SPE)-based Li-batteries is the functionality of the electrolyte decomposition layer that is spontaneously formed at the Li metal anode. A deeper understanding of this layer will facilitate its improvement. This study investigates three SPEs – polyethylene oxide:lithium tetrafluoroborate (PEO:LiBF_4_), polyethylene oxide:lithium bis(oxalate)borate (PEO:LiBOB), and polyethylene oxide:lithium difluoro(oxalato)borate (PEO:LiDFOB) – using a combination of electrochemical impedance spectroscopy (EIS), galvanostatic cycling, *in situ* Li deposition photoelectron spectroscopy (PES), and *ab initio* molecular dynamics (AIMD) simulations. Through this combination, the cell performance of PEO:LiDFOB can be connected to the initial SPE decomposition at the anode interface. It is found that PEO:LiDFOB had the highest capacity retention, which is correlated to having the least decomposition at the interface. This indicates that the lower SPE decomposition at the interface still creates a more effective decomposition layer, which is capable of preventing further electrolyte decomposition. Moreover, the PES results indicate formation of polyethylene in the SEI in cells based on PEO electrolytes. This is supported by AIMD that shows a polyethylene formation pathway through free-radical polymerization of ethylene.

## Abstract 2

A limiting factor for solid polymer electrolyte (SPE)-based Li-batteries is the functionality of the electrolyte decomposition layer that is formed at the Li metal anode during battery operation.

## Graphical abstract

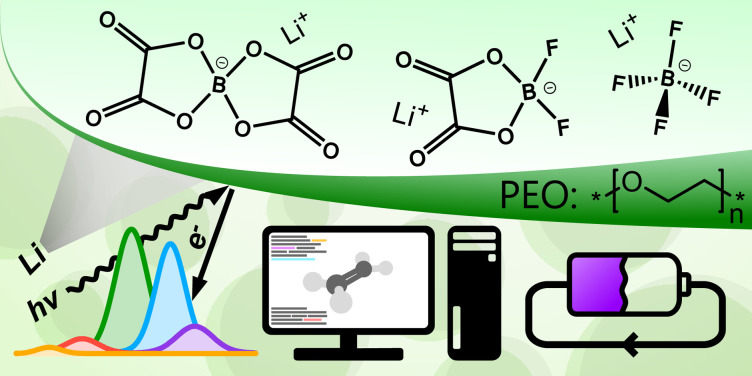

## Introduction

An ever-increasing demand on energy storage performance, both in specific energy (per mass) and energy density (per volume), has been pushing the research community to continuously find new – and improve existing – battery chemistries in order to improve these metrics. An example of such chemistries is solid polymer electrolytes (SPEs) used with either Li-metal as the anode or possibly even anode-free cell concepts.^1–4^ A Li-battery based on SPEs is also an interesting alternative to liquid electrolyte LIBs, due to a much lower flammability of the electrolyte, making it safer.^5^ However, even though Li-metal or anode-less SPE-based Li-batteries are attractive concepts, they are still limited by a finite number of charge cycles due to unfavorable side reactions and a loss of active material.^6,7^

One decisive factor for the stability of conventional LIBs based on liquid electrolytes is the functionality of its solid electrolyte interphase (SEI).^8^ Decomposition of the electrolyte occurs at the anode–electrolyte interface when operating at low potentials outside of its stability window, and ideally this decomposition will lead to the formation of an SEI. The composition of the SEI can vary drastically depending on the composition of the electrolyte and type of anode, but ideally the SEI layer is an electronically insulating but ionically conducting barrier between the electrolyte and the electrode. In this way it limits the continuous consumption of the electrolyte throughout the operation of the battery while still allowing for the transfer of ions between electrolyte and anode.^8–10^ This is why formation of a stable SEI is crucial to obtain batteries with good performance and a long cycle life.

For SPE-based batteries, as compared to conventional LIBs there is less understanding of the composition and function of the SEI as compared to conventional LIBs. In general, it is understood that the SEI plays a very important part in the performance of SPE-based cells owing to the large interfacial resistance in these,^11,12^ and there exists a small collection of studies regarding the interphases in SPE-based cells. These studies have investigated the effects of both polymer and salt, and even contaminants like water, on the composition of the interphase.^12–17^ However, more studies on a larger platform of materials are needed in order to better understand the formation of the SEI and its composition, and one important aspect that needs to be investigated in this context is the Li salt used in the electrolyte.

**Fig. 1:**
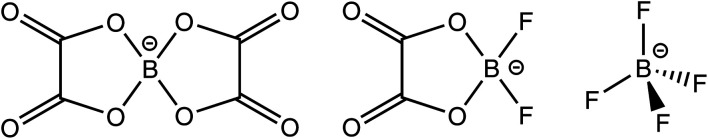
BOB, DFOB and BF_4_^−^ anions.

Lithium hexafluorophosphate (LiPF_6_) is the most commonly used Li salt in conventional LIBs, and one reason for using this salt is that it contains fluorine, which is a decisive element in the formation of a stable and functional SEI. For example, one of the SEI components often seen is lithium fluoride (LiF), which is generally considered a favorable part of the SEI and originates from decomposition of fluorinated salts. For similar reasons, other fluorine-containing Li salts have been considered for LIBs, such as lithium tetrafluoroborate (LiBF_4_) and lithium bis(trifluoromethanesulfonyl)imide (LiTFSI).^18–20^ LiTFSI has proven successful in most SPE chemistries, and has been the standard for decades,^17,21–23^ however several new salts for SPEs have been highlighted in recent years.^24–26^ In this context, lithium bis(oxalato)borate (LiBOB) is a salt that has shown interesting properties with regards to creating a stable SEI layer in LIB cells using a graphite anode and liquid electrolyte,^27–34^ and has also shown promising results in terms of conductivity when used in combination with polyethylene oxide (PEO) in SPEs.^35,36^ A study on the reduction of LiBOB along with several studies on the SEI layer formation has shown oxalates, carbonates, semi-carbonates, and crosslinked oligomeric borates to be among the decomposition products.^29,30,37^ Lithium difluoro(oxalate)borate (LiDFOB), in turn, is a fluoride-containing borate salt, which has shown good properties for creating a stable SEI layer in liquid LIBs,^19,33,38–40^ and also promising results in anode-free cells.^41^ LiDFOB, which can be seen as an intermediate of LiBF_4_ and LiBOB (Fig. 1), shares properties of both salts.^38^ Its decomposition products in LIBs constitute a combination of those of LiBOB and those of LiBF_4_, mainly Li_*x*_BF_*y*_ and LiF.^19,37^ Species containing C=O, B–F and B–O groups have also been suggested as decomposition products.^39^

In a previous study, we used *in situ* Li deposition to characterize three SPE systems, all using LiTFSI but solvated in different polymers.^42^ Using this technique, Li is deposited directly onto the SPE material, while the changes to the surface chemical composition are observed using soft X-ray PES. This simulates the plating of Li occurring in Li-metal and anode-free systems, and gives a picture of the initial SEI composition, without the need of separating the SPE from the electrode post-mortem. While this previous study investigated three polymers containing the same Li salt, we here instead focus on the effect of the salt. Here, we chose the SPE polymer host PEO, due to its apparent stability in the previous *in situ* Li deposition PES study. It also possesses a comparatively simple core-level spectra (only one peak each for C 1s and O 1s), making analysis of the salt decomposition products easier than for other polymers. Furthermore, we build upon our previous experiences of employing *ab initio* molecular dynamics (AIMD) simulations in order to gain a greater understanding of the Li|SPE interface,^43^ and we have also combined this with charge analysis in order to aid the interpretation of PES spectra.^44^

In this study, the *in situ* Li deposition PES method is employed to investigate the anode interface of PEO-based SPE films containing LiBOB, LiBF_4_, and LiDFOB salts (PEO:LiBOB, PEO:LiBF_4_ and PEO:LiDFOB). The charge analysis methodology from AIMD is used as a complement to the PES data. Electrochemical impedance spectroscopy (EIS) and galvanostatic cycling of Cu|SPE|LFP cells with electrolytes are used to connect the SEI composition obtained from the combined AIMD and PES analysis to the electrochemical performance of the SPEs. The results show that a relatively small amount of decomposition of the Li salt can still result in a high capacity retention during cycling. The results also show that Li deposition can lead to polymerization at the anode interface.

## Materials and methods

### Polymer electrolyte preparation for electrochemical characterization

Polymer electrolytes were prepared in an Ar-filled glovebox (H_2_O < 1 ppm, O_2_ < 1 ppm) by dissolving the polymer (PEO, Sigma Aldrich, average *M*_v_ ∼2 000 000) and the Li^+^ salts (LiTFSI, BASF; LiDFOB, Sigma-Aldrich; LiBOB, ChemMetal; LiBF_4_, Sigma-Aldrich) in anhydrous acetonitrile (ACN, Sigma-Aldrich) overnight, followed by casting in PTFE moulds to create films for electrochemical characterization or on stainless steel sample plates (Scienta Omicron) to get SPE films for PES characterization. Complete removal of the solvent was ensured by using a casting protocol described previously, in which the pressure is gradually lowered to less than 2 mbar, and the temperature is kept at 30 °C for 20 h and then raised to 60 °C for 40 h.^45^ The Li^+^ salt content was kept constant at 25 wt%. After this, the sample plates with films for PES characterization were vacuum sealed and mounted in the instrument without direct contact to air. The polymer films for electrochemical characterization were hot-pressed at 80 °C applying a pressure of 20 MPa to control the thickness at 100 μm ± 10 μm and then punched. All the Li^+^ salts were vacuum dried at 120 °C for 48 h before use.

### Electrode preparation

LiFePO_4_ (LFP) electrodes were prepared by ball milling the LFP powder active material (Tobmachine) with carbon black (CB, C65, Imerys) and polyethylene oxide (PEO 400k, Sigma Aldrich) as binder, with the ratio of LFP/CB/binder at 70 : 15 : 15, using anhydrous acetonitrile as solvent. The electrode slurry was coated on aluminum foil with a doctor blade gap of 150 μm. The electrodes were dried overnight at room temperature and finally punched and dried in a vacuum oven (Buchi) at 120 °C for 5 h and stored in protected atmosphere inside an Ar-filled glovebox (H_2_O < 0.5 ppm, O_2_ < 0.5 ppm). The active mass loading of the as prepared electrode was around 1.5–2.0 mg cm^−2^.

For the current collector, a 30 μm thick Cu foil was used. The foil was punched and underwent a brief cleaning process involving diluted hydrochloric acid (HCl 5%, Sigma Aldrich) to obtain a fresh surface. It was then thoroughly rinsed with deionized water and ethanol, carefully dried in a vacuum oven at 60 °C and stored in protected atmosphere to avoid further oxidation of the surface.

### Electrochemical characterization

For the electrochemical characterization methods, a PEO-based SPE containing LiTFSI was included in order to compare the obtained results to a commonly used salt in SPEs.^22,23^ The ionic conductivity of the SPEs was investigated using electrochemical impedance spectroscopy (EIS). EIS measurements were conducted between 10 MHz and 1 Hz applying a single-wave potential perturbation with an amplitude of 10 mV around the open-circuit voltage (OCV). The experiments were performed using a Schlumberger 1260 frequency-response analyzer in the temperature range 22–80 °C. One day prior to the EIS characterization, the coin cells (CR2025, MTI) containing the SPE films were annealed at 80 °C for 1 h to ensure optimal contact with the stainless steel blocking electrode. The bulk resistance of the polymer electrolytes was determined by fitting a Debye circuit using ZView software (Scribner Associates).

CR2032 coin cells for galvanostatic cycling characterization were assembled in anode-free configuration using a Cu current collector as anode and an LFP electrode as cathode, with the SPE film sandwiched in between. Galvanostatic cycling tests were performed using an Arbin BT-2043. Constant current cycling tests were performed at 0.1C at a temperature of 60 °C, with an upper and lower cutoff voltage of 4.0 V and 2.7 V, respectively. The cells were allowed to rest at OCV conditions for 10 h at 60 °C prior to testing to ensure good interfacial contact.

### DFT calculations

All DFT calculations and AIMD simulations were performed in the Vienna *Ab initio* Simulation Package (VASP) 6.1.1 (ref. 46 and 47) with the projector augmented wave (PAW) method.^48^ The Perdew–Burke–Ernzerhof (PBE)^49^ generalized gradient approximation (GGA) functional was used to describe exchange and correlation interactions. The van der Waals correction was included using the DFT-D3 method. The energy and force convergence were set to 10^−4^ eV and 0.01 eV Å^−1^, respectively, and the energy cut-off was set to 500 eV. A 1 × 1 × 1 gamma *k*-point mesh was used to sample the Brillouin zone in the AIMD simulation. For the climbing image-nudged elastic band (CI-NEB) and frequency calculations, a 3 × 3 × 1 *k*-point mesh and a tighter energy convergence value (10^−5^ eV) was used for frequency calculation. The transition states along the LiBF_4_ decomposition pathway were searched using the CI-NEB method.^50^ Frequency calculations were performed to examine the stable adsorption structures and transition states. All energies were corrected to Gibbs free energies at 298 K using eqn (1):1
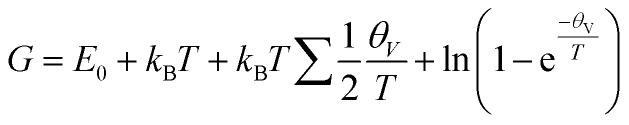
where *G* is the Gibbs free energy of systems, *E*_0_ is the electronic energy of systems, *k*_B_ is the Boltzmann constant, *T* is the temperature (298 K), and *θ*_V_ is the vibrational temperature.

### *Ab initio* molecular dynamics

The simulation cell for the electrolytes was constructed using the amorphous cell module of the Materials Studio code. This module generated a packing structure of PEO chains and salt molecules based on the COMPASS II force-field. The concentration of Li salt was maintained at ∼25% for all three SPEs, and the compositions of the SPEs are detailed in Table S1.† Simulation models of the three SPEs are depicted in Fig. S1a–c.† To build the interface model, the three SPEs were placed on the Li (100) surface. Subsequently, we conducted a classical MD pre-equilibrium process using the Forcite module with the COMPASS II force-field to obtain a reasonable initial configuration of interfaces. The temperature was set to 400 K for 10 ps (NVT ensemble). The models of the three SPEs on Li (100) surfaces are illustrated in Fig. S1d–f.† To investigate the Li|SPE interfacial reactions, AIMD simulations were conducted using the NVT ensemble, with the temperature set to 400 K for the primary simulation focusing on the interfacial reaction between the electrolyte and Li anode. Additionally, a temperature of 600 K was employed for the Li-atoms pre-equilibrium step, corresponding to the relaxation of plated Li atoms. The calculation procedure followed our previous work.^44^ The AIMD simulation started with a 5 ps duration to simulate the SPE on the static Li (100) anode surface. Subsequently, ten Li atoms were added close to the anode surface to mimic the Li plating process on the Li anode surface. Following the addition of extra Li atoms, a Li-atoms pre-equilibrium step was performed to facilitate the relaxation of the additional Li atoms. The primary AIMD simulation was then extended for an additional 3 ps. This Li nucleation process was iteratively repeated three times, involving the addition of ten Li atoms, a pre-equilibration step, and an additional 3 ps AIMD simulation each time. Furthermore, a 10 ps AIMD simulation was conducted to investigate the pure SPE, representing the polymer before any reaction with Li, which was utilized for the charge analysis.

### Charge analysis

Ten configurations from the last 500 fs of the AIMD simulations were extracted at intervals of 50 fs, including those of the SPE bulk and the Li|SPE interface. Then, the Bader charge^51–53^ of each atom was calculated for each configuration, and the charge distribution was presented using a Gaussian function for each chemical environment.

### Binding energy calculation

The binding energies of Li-ion on the PEO chain and salt anions were computed using DFT within the Gaussian 09 package. The Minnesota hybrid meta-GGA function, M06-2X, and 6-311+G(d,p) basis sets were applied. To model the complexes in the PEO medium, the SMD implicit solvent model with a dielectric constant of 7 was employed.^54,55^ The binding energies of the complexes were determined by subtracting the sum of the energies of individual Li-ion and polymer chains or salt anions from the total energy of the complexes. This energy calculation includes both the electronic energy and the zero-point energy associated with vibrational motions.

### Photoelectron spectroscopy and *in situ* Li deposition

PES measurements were carried out at the LowDosePES^56^ station on the PM4 (ref. 57) beamline at the BESSY II electron storage ring operated by the Helmholtz-Zentrum Berlin für Materialien und Energie. The end-station is equipped with a high transmission angular-resolved time-of-flight (ArTOF) spectrometer with a ±30° acceptance angle, which is optimized for measurements of radiation sensitive samples. The pressure in the analysis chamber was always kept in or below the low 1 × 10^−9^ mbar region during measurements but almost always in the 1 × 10^−10^ mbar region.

The B 1s, C 1s, O 1s, and F 1s core-levels were measured. To get a consistent depth of analysis, the photoelectron kinetic energy was kept between 300 and 310 eV. The photon energies therefore were set at 500, 600, 845, and 1000 eV, respectively. The C 1s core-level was recorded for each photon energy every time the photon energy was changed and subsequently used as internal binding energy reference. For calibration, the position of the PEO polymer peak in the C 1s spectra was set to 286.6 eV in binding energy and related core level spectra were then adjusted accordingly. While changing photon energy the measurement spot on the sample was moved in order to avoid measuring the effects of possible radiation damage to the sample. All core-levels were measured before and after a 15 min Li deposition step using a resistively heated Li dispenser (S.A.E.S group), at 7.3 A and 4.2–4.4 V (a method developed by Wenzel *et al.*^58^). During Li deposition, the pressure in the deposition chamber was in the range of 10^−8^ mbar. All PES data treatment (energy calibration, curve fitting) was done using Igor pro version 9.0.1.2 using the Spectral Analysis by Curve Fitting (SPANCF) package by Edwin Kukk.

## Results and discussion

In this study three different SPEs, PEO:LiBF_4_, PEO:LiBOB, and PEO:LiDFOB, are investigated. In order to establish their performance, the electrochemical characterization of the SPEs through galvanostatic cycling and ionic conductivity measurements is presented. A combination of AIMD and PES with *in situ* Li deposition is used to investigate the Li|SPE interface, to characterize the initial decomposition at the Li|SPE interface and correlate this to long-term electrochemical stability of the cells.

### Cycling and conductivity results

**Fig. 2:**
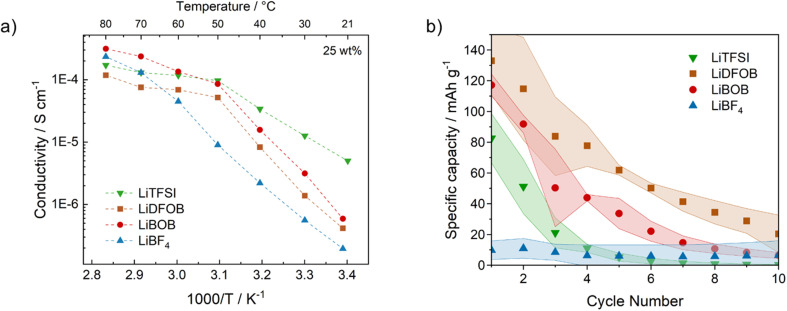
(a) Ionic conductivity as a function of temperature of PEO:salt SPEs, salt concentration fixed at 25 wt%. (b) Specific capacity of anode-free batteries (Cu *vs.* LFP, N/P = 0) containing different PEO:salt SPEs. Test performed at 60 °C, 0.1C (≈40 μA cm^−2^), in the voltage interval 2.7–4.0 V.

Electrochemical characterization was performed on the three SPE materials, PEO:LiBF_4_, PEO:LiBOB, and PEO:LiDFOB, with PEO:LiTFSI as a reference material. First, impedance measurements were performed to evaluate the bulk resistance of the SPEs. The total ionic conductivity values calculated from these bulk resistances are shown in Fig. 2a, at temperatures from 22 to 80 °C. The conductivities range from 10^−7^ to 10^−3^ S cm^−1^ and show good agreement with previously published results.^59–61^ In particular, a rapid increase in ionic conductivity is shown until the temperature reaches a value around 50 °C, indicative of melting of the semi-crystalline PEO matrix with faster Li^+^ ion transport as a result of the increase in amorphous content of the polymer.^62–64^ The benchmark sample, PEO:LiTFSI, shows the highest ionic conductivity at lower temperatures (5 × 10^−6^ S cm^−1^), but PEO:LiDFOB and PEO:LiBOB show comparable results at temperatures higher than 50 °C (>10^−4^ S cm^−1^). This indicates that LiTFSI is more efficient than the other salts at amorphizing the PEO host polymer matrix, which is expected due to the size and flexibility of the anion. PEO:LiBF_4_, however, shows the lowest values below 60 °C out of all salts, suggesting a poor suitability for LiBF_4_ as the main conductive source in PEO-based SPEs. To elucidate Li-ion transportation within PEO-based SPEs, we calculated the binding energy of Li-ion on the PEO chain and salt anions, as outlined in Table S2,† with the corresponding optimized structures presented in Fig. S2.† The coiled-type binding between a PEO chain and Li-ion yields the maximum number of oxygen atoms for coordinating Li-ion, resulting in a Li binding energy of −3.00 eV. Considering the highest binding energies of Li-ion on BF_4_^−^, BOB, and DFOB salt anions, which are −1.86, −1.68, and −1.89 eV, respectively, the coiled-type binding can be viewed as a driving force for the dissolution of the Li salts. However, in other configurations than the coiled-type the binding energy is lower, as exemplified by the anchored-type binding with a binding energy of −0.86 eV. This suggests that the PEO chain can modulate its binding abilities by adopting different orientations. The dynamics of the Li ion binding leads to the absence of a fixed barrier for ion transport, and as a result to a non-Arrhenius behavior in Li-ion migration. It is important to note that the specific details of Li-ion transportation within each electrolyte lie outside of the scope of this investigation, and for which AIMD would be too resource intensive.

Anode-free cells (Cu *vs.* LFP) were assembled to test the behavior of the different PEO:salt compositions during cycling (cycling curves shown in Fig. S3–S6†). From Fig. 2b it is clear that, despite having higher ionic conductivity, the PEO:LiTFSI electrolyte shows worse cycling performance than PEO:LiBOB and PEO:LiDFOB. In particular, the 1st cycle capacity was 82 mA h g^−1^ for PEO:LiTFSI while PEO:LiBOB and PEO:LiDFOB reached values of 117 mA h g^−1^ and 133 mA h g^−1^ respectively. In terms of capacity retention, the PEO:LiDFOB system exhibited the best results, maintaining a consistent coulombic efficiency of 80% throughout the cycling (Fig. S7†). Compared to the other Li^+^ salts, especially LiTFSI used as a reference, this clearly indicates that in an anode-free configuration the ionic conductivity is not a decisive parameter for the long-term performance. As will be shown below, the results rather suggest that the most important parameters are the functionality of the SEI layer, and the reversibility of the Li metal plating and stripping processes, both heavily influenced by the nature of the Li salt anion.

### *Ab initio* molecular dynamics and suggested decomposition products

The interfacial reactions of PEO:LiBF_4_, PEO:LiBOB, and PEO:LiDFOB SPEs on a Li anode surface were explored by means of AIMD simulations. The simulations of all three interfaces were performed by first allowing the SPEs to reach a relaxed state on the Li surface. After this, Li atoms were added and allowed to react, following a method bench-marked in previous work.^44^ The addition of Li was done three times in 3 ps intervals. The reaction products were noted after the final Li addition, and the calculated charges were used to aid the PES interpretation. Energy fluctuations for each system are illustrated in Fig. S8,† and the total energy tends to be constant at the end of each stage to ensure these systems are close to equilibrium. Building upon our previous work, it is evident that chain length does not significantly impact the electronic properties of polymers, such as frontier orbital energies, density of states, and redox potential.^54,65,66^ This is attributed to the aliphatic backbone of PEO, composed of sp^3^ carbons, preventing the delocalization of electrons. For computational efficiency, we utilized a PEO chain with six monomers, which retains reliable electronic chemical properties representative of a real polymer chain. However, it is important to note that the segmental dynamics of polymer chains are strongly influenced by chain length. This suggests that simulations of ion transport need to consider a sufficiently long PEO chain. The physical and mechanical properties of PEO electrolytes, which go beyond the capabilities of AIMD simulations, are beyond the scope of this study.

#### PEO:LiBF_4_

**Fig. 3:**
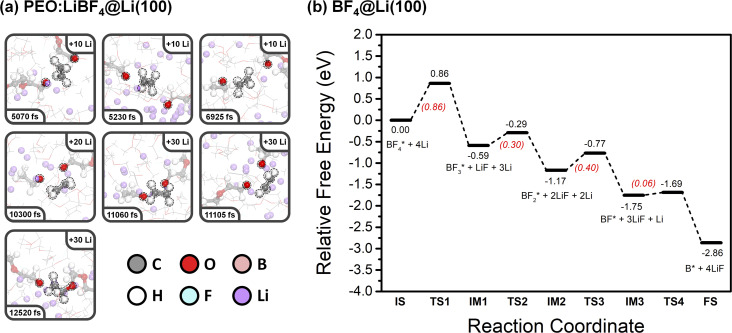
(a) Snapshots of the PEO:LiBF_4_ electrolyte on a Li (100) surface extracted from AIMD simulations at various points in time. (b) Calculated potential energy profile for the decomposition of BF_4_^−^ on a Li (100) surface (* denotes adsorbed species).

In PEO:LiBF_4_, the first PEO chain decomposed into two Li alkoxide and one ethylene units *via* two C–O bond cleavages at 5070 fs (first Li nucleation stage), as Fig. 3a shows. This PEO decomposition mechanism is similar to what we have observed previously for the PEO:LiTFSI system.^44^ Following this, other PEO chains decomposed into the same products (Li alkoxide and ethylene) at 5230, 6925, 10 300, 11 060, and 11 105 fs by the same mechanism.

An incomplete decomposition of one PEO chain occurred at 12 520 fs, producing one Li alkoxide and one ROC_2_H_4_ fragment. This incomplete decomposition is most likely due to the insufficient number of Li atoms added in our simulation. In a Li metal battery, it is expected that the large amount of Li atoms plated on the anode during charging would ensure that this ROC_2_H_4_ intermediate further converts to Li alkoxide and ethylene. A fraction of the ethylene molecules formed from PEO decomposition further reacts with Li atoms and forms Li ethylene complexes (Li_2_C_2_H_4_). As these are difficult to observe in the snapshots, they are highlighted at the final state of all three SPE systems in the ESI (Fig. S9).† Interestingly, the decomposition of the BF_4_^−^ anion was not observed in the simulation. To explore this mechanism, the corresponding free-energy potential diagram was calculated using the climbing image nudged elastic band (CI-NEB) method, as shown in Fig. 3b. The four B–F bond cleavages are all exergonic reactions, and the rate-determining step is the first B–F bond cleavage (Δ*G*^‡^ = 0.86 eV). This relatively high barrier can help explain the stability of the BF_4_^−^ anion during the AIMD simulation, even though the PES experiments show breakdown of the BF_4_^−^ anion, as will be discussed later. Optimized geometries of the initial state, intermediates, transition states, and the final state of BF_4_^−^ decomposition are shown in Fig. S10.†

#### PEO:LiBOB

**Fig. 4:**
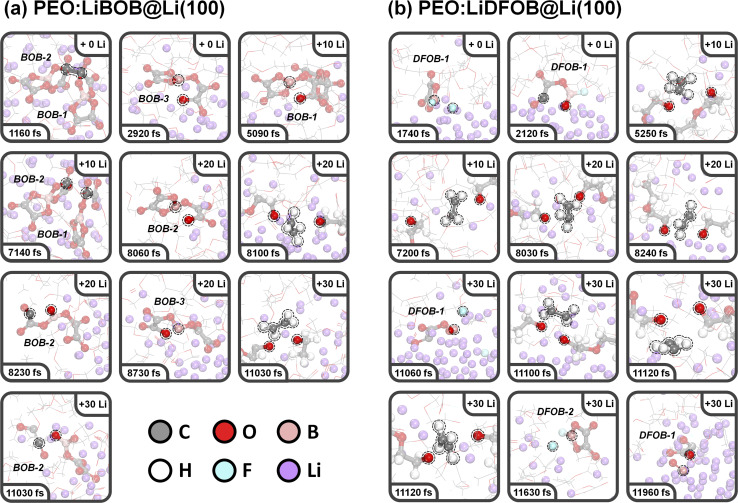
Snapshots of the (a) PEO:LiBOB and (b) PEO:LiDFOB electrolyte on a Li (100) surface extracted from AIMD simulations at various timepoints.

Snapshots of the PEO:LiBOB SPE on Li (100) are shown in Fig. 4a. Here, we found PEO decomposing at 8100 and 11 030 fs *via* the same mechanism as for the PEO:LiBF_4_ SPE. For PEO:LiBOB, however, this occurs later, and fewer PEO molecules are decomposed compared to PEO:LiBF_4_ (2 *vs.* 6), which is attributed to a relatively high reactivity of the BOB anion. The fast BOB reductive decomposition consumes most of the added Li atoms, and the oxygen atoms of BOB bind tightly with the Li ion. Together, this leads to a slower decomposition of PEO (in the second and third Li nucleation stages). Looking at the first and second BOB anions, labeled BOB1 and BOB2 in Fig. 4a, the AIMD simulations show that two BOB anions can undergo dimerization (*via* C–C coupling, 1160 fs). Following this, BOB1 experiences a ring-opening reaction at 5090 fs, followed by a de-dimerization (the BOB1–BOB2 bond is broken) at 7140 fs, resulting in a BC_4_O_8_ species (labeled as B^(3)^ in the charge analysis; B^(*n*)^ indicates B with *n* coordination). BOB2 experiences two ring-opening reactions (O–B/C–O bond cleavage at 8060/8230 fs), and then decomposes into C_2_O_2_ and BC_2_O_6_ (also labeled B^(3)^) species at 11 030 fs. The third BOB anion, BOB3 (that did not partake in dimerization), experiences a similar B–O-breaking ring-opening reaction to produce a BC_4_O_8_ species at 2920 fs, and then yet another similar ring-opening reaction to form BC_4_O_8_ (B^(2)^) finally. Thus, in this simulation, the decomposition of three out of four BOB anions was observed, and the fourth BOB fragment was found to be buried in the SEI layer and highly surrounded by Li. This result demonstrates the high reactivity and Li affinity of the BOB anion, which in turn leads to a relatively lower degree of PEO decomposition.

#### PEO:LiDFOB

Snapshots of the PEO:LiDFOB SPE on Li are shown in Fig. 4b. In this system, PEO decomposed at 5250, 7200, 8030, 8240, 11 100, and 11 120 fs, showing similarities with the reactivity of PEO in PEO:LiBF_4_. The first DFOB anion decomposed to produce a B^(3)^ species, BFC_2_O_4_, *via* B–F bond cleavage at 1740 fs; the fluorine adsorbs to the surface as a single LiF complex. A subsequent ring-opening reaction and a second B–F breaking of BFC_2_O_4_ happened at 2120 and 11 060 fs, respectively. After complete defluorination, a B^(2)^ species, BC_2_O_4_, formed and further rapidly converted to BO and C_2_O_3_ fragments at 11 960 fs. The second DFOB anion undergoes one defluorination and forms a BFC_2_O_4_ fragment in the third Li nucleation stage (11 630 fs). Based on this observation, the simulations show that two out of five DFOB anions decomposed, implying that DFOB anions are more stable than BOB anions.

**Fig. 5:**
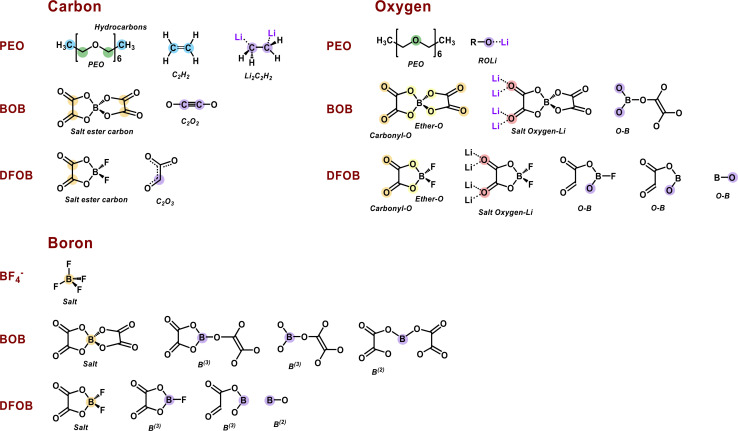
Chemical structures and corresponding bonding environment of proposed intermediates and SEI components from the PEO host polymer, BF_4_^−^, BOB, and DFOB anions in the different SPE@Li(100) systems. Charges are not denoted here since the charges for each species is detailed in the charge analysis in the next section.

A summary of the step-by-step interfacial reactions in three SPEs can be found in Table S3.† The intermediates and SEI components from these AIMD calculations are shown in Fig. 5. The color of the atoms in every chemical structure corresponds to different chemical environments that will be further discussed in conjunction with the experimental PES results below.

### Photoelectron spectroscopy and charge analysis

PES measurements in combination with *in situ* Li deposition were used to obtain a picture of the interface during the first charge cycle of anode-free SPE-based Li batteries with PEO:LiBF_4_, PEO:LiBOB, and PEO:LiDFOB as the SPEs. This method produces a collection of spectra before and after Li deposition. In this section C 1s, O 1s, B 1s, and F 1s spectra are presented. Li 1s spectra are found in Fig. S11 and S12,† as the low signal to noise ratio prevented any useful interpretation. Charge analysis results for the calculated decomposition products from the previous section are presented along with the PES spectra in this section. In order to facilitate the comparison, the charge analysis results are presented with the electron charge |*e*| as unit corresponding to eV in the binding energy spectra. It is assumed that the decomposition in the simulations at a temperature of 400 K is comparable to room temperature at which decomposition in the PES experiments took place. In both the PES and charge analysis spectra, green represents PEO or the SPE in general, if deconvolution was impossible. Blue represents hydrocarbons. Orange and yellow represent the unaffected salt anions in the SPE. Red represents salt anions that have not been decomposed by Li but are still influenced by its presence. A purple color represents a decomposition product.

#### C 1s

**Fig. 6:**
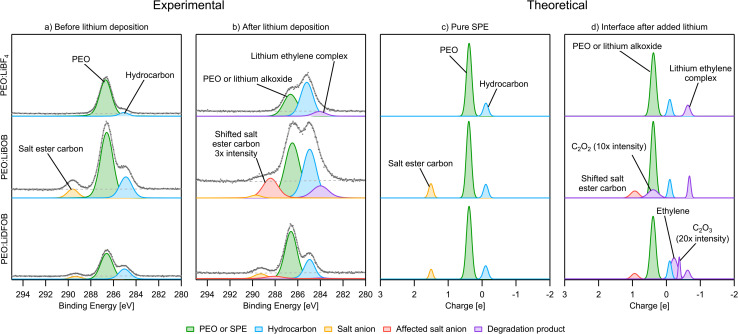
(a) PES C 1s spectra before Li deposition. (b) PES C 1s spectra after deposition. (c) Charge analysis spectra for carbon of the pure SPE. (d) Charge analysis spectra for carbon of the Li|SPE interface. The intensity of some peaks have been increased in order to improve visibility. PES spectra intensity is not normalized.

The C 1s PES spectra from before deposition (Fig. 6a) are as expected from the compositions of the samples. For the pristine sample of all SPEs, the PEO (C–O) carbon is set to 286.6 eV, and some surface hydrocarbon (C–H/C–C) is found at 285.0 eV. The carbon specific to the BOB/DFOB anion is found at 289.6 eV for PEO:LiBOB and 289.3 eV for PEO:LiDFOB. These binding energy positions are also consistent with the charge distribution plot of the pure SPEs (Fig. 6c). Two peaks in the charge distribution plot of carbon (Fig. 6c) belonging to the PEO chain at +0.4 and −0.1|*e*| can be seen for all SPE systems. These correspond to C–O and hydrocarbon (C–C/C–H), respectively. In the PEO:LiBOB and PEO:LiDFOB SPEs, the additional peak (+1.5|*e*|) corresponds to the ester carbon in the BOB/DFOB anion. It should be noted that the hydrocarbon peak seen in the PES spectra before deposition (Fig. 6a) does not originate from the same source as the hydrocarbon peak in the charge analysis spectra before the addition of Li (Fig. 6c). In the PES results, the hydrocarbon peak most likely originates dominantly from surface contamination, while the hydrocarbons in the calculations originate from the end groups of the polymer. Due to the short chain length used in the AIMD simulations, the end groups are visible in the charge analysis, while in the real polymer, the main chain makes up most of the polymer, meaning that the concentration of end groups is extremely low, rendering the end groups imperceptible in the PES spectra.

**Fig. 7:**
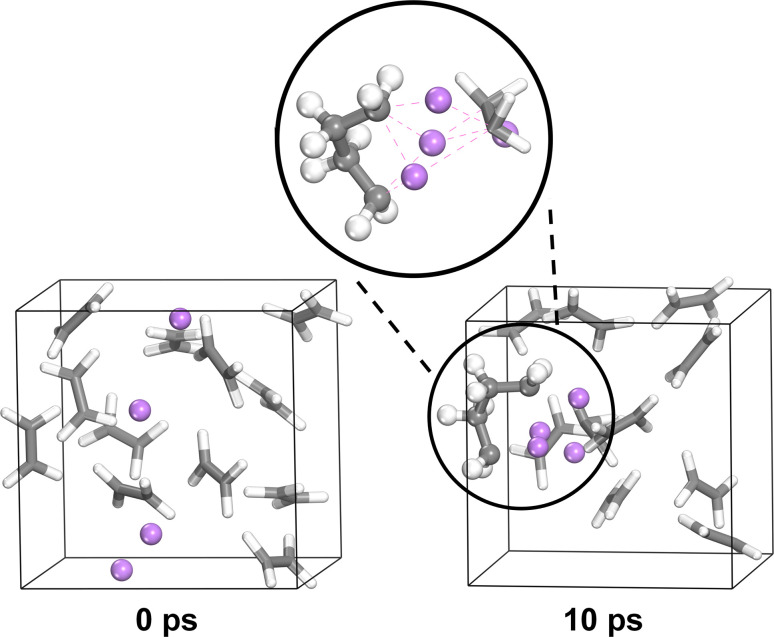
AIMD simulation of the polymerization of ethylene into polyethylene.

After Li deposition (Fig. 6b), the formation of more hydrocarbons in the PES spectra becomes apparent for PEO:LiBF_4_ and PEO:LiBOB. This was previously seen as surface hydrocarbons remaining at a constant intensity while the other carbon-containing species decreased in intensity.^42^ In the carbon charge analysis spectrum, the main PEO decomposition products are ethylene molecules (C_2_H_4_, −0.2|*e*|) and Li ethylene complexes (Li_2_C_2_H_4_, −0.7|*e*|). Ethylene has a binding energy similar to that of hydrocarbons, but being a gas at room temperature and atmospheric pressure, it will certainly leave the surface region out into the vacuum of the deposition chamber before the PES analysis can take place. This is why ethylene is not expected in the experimental PES spectra. With this in mind, it is worth reconsidering the reason for the increase in the hydrocarbon peak. Another candidate for this peak, considering the abundance of ethylene and Li ethylene complexes in the AIMD results, is polyethylene (PE). In order to confirm the viability of the polymerization of ethylene and Li ethylene complexes into PE, a 10 × 10 × 10 Å^3^ simulation box containing 12 ethylene molecules and 4 Li atoms was run using AIMD (Fig. 7). The initiation of ethylene polymerization into a C_4_H_8_ oligomer was observed at 7960 fs by donation of an electron from the coordinated Li to the ethylene, indicating a free-radical polymerization mechanism. This suggests that it is indeed possible for the polymerization to take place. The polymerization reaction is further supported by the peak at 284.0 eV in the PES spectra of PEO:LiBF_4_ and PEO:LiBOB, attributed to the Li ethylene complexes found in the AIMD simulations.

With regards to the stability of the salts, there is no carbon in LiBF_4_, so the C 1s PES results are focused on PEO:LiBOB and PEO:LiDFOB. Overall, the C 1s spectrum of PEO:LiDFOB changes considerably less after deposition compared to PEO:LiBOB. The only discernible difference after Li deposition on PEO:LiDFOB is a small peak appearing at ∼288 eV. Previous studies with these salts have found Li carbonate and Li oxalate as decomposition products, as detailed in the introduction. In the C 1s spectra of PEO:LiBOB and PEO:LiDFOB after deposition (Fig. 6), no clear peak for Li carbonate is found for either salt, but the peak at 288.4 eV in the PEO:LiBOB system and 288 eV in PEO:LiDFOB might be interpreted as Li oxalate according to Michan *et al.*^67^ There are, however, other candidates for this peak found in the AIMD simulations. When the BOB/DFOB anion is highly surrounded by ionic Li, the ester carbon peak shifts toward +0.9|*e*| (Fig. 6d). The presence of Li oxalate is discussed further in the O 1s section. In addition to the decomposition compounds previously discussed, the AIMD calculations show that incomplete decomposition of the BOB and DFOB anions produces C_2_O_2_ (+0.4|*e*|) and C_2_O_3_ (−0.4|*e*|) fragments, respectively. These fragments are deemed unlikely to be a prominent part of the decomposition layer seen in the PES results due to their unstable nature. It is impossible to completely rule these out, however, as their peak intensity in a PES spectrum would be very low. Also, in the case of C_2_O_2_, its peak in the charge analysis overlaps completely with PEO, meaning that it would be impossible to identify it clearly in any PES spectra.

#### O 1s

**Fig. 8:**
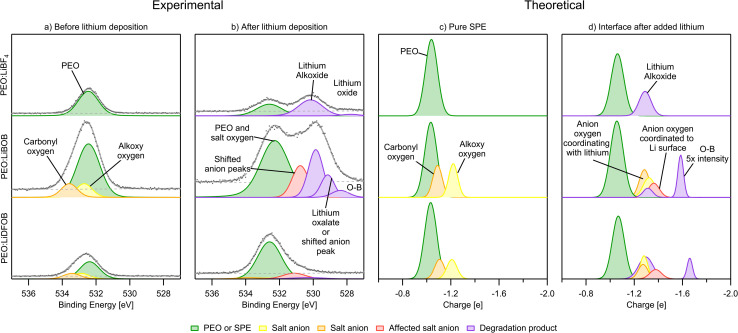
(a) PES O 1s spectra before Li deposition. (b) PES O 1s spectra after deposition. (c) Charge analysis spectra for oxygen of the pure SPE. (d) Charge analysis spectra for oxygen of the Li|SPE interface. The intensity of some peaks have been increased in order to improve visibility. PES spectra intensity is not normalized.

The O 1s PES spectra show just one feature in all three samples before Li deposition, as seen in Fig. 8a. For PEO:LiBF_4_ this is expected since there is only one oxygen environment in PEO. For the other two systems, two additional peaks originating from the salt are expected. Using the relative intensity of the PEO and salt peak from the C 1s spectra, the corresponding relative intensity between the two salt peaks and the PEO peak in the O 1s spectra of PEO:LiBOB and PEO:LiDFOB was calculated. This, together with the relative binding energy position obtained from reference spectra from the pure salts (Fig. S13 and S14†) and the PEO:LiBF_4_ O 1s PEO peak, were used as input parameters for curve fitting the PES spectra. In the AIMD simulations, there is a main peak (green color) for all three SPEs that originate from the ether oxygen atoms in the PEO backbone at −1.05|*e*|. For the PEO:LiBOB and PEO:LiDFOB systems, two equal-area peaks at −1.1 and −1.2|*e*| represent carbonyl (C=O) and alkoxy (C–O–B) oxygens, respectively, in the oxalate group. An interesting observation is that the relative order (in terms of binding energy/charge) of carbonyl and alkoxy oxygens in boron chemicals is the opposite of carboxylate esters.^68^ This phenomenon can be rationalized by the lower electronegativity of boron compared to carbon so that the alkoxy oxygen gets a higher electron density in the BOB/DFOB anion than the alkoxy oxygen in a carboxylate ester group. Another interesting observation is that the peaks of the salt in the PES spectra of both PEO:LiBOB and PEO:LiDFOB are at a higher binding energy than the PEO oxygen, while they have a lower charge compared to the PEO peak in the charge analysis. Assuming that PEO should have similar binding energy peak position in all SPEs means that the binding energy positions of the salt ether and carbonyl oxygen peaks must be at higher binding energies compared to the PEO peak in both PEO:LiBOB and PEO:LiDFOB. This is also supported by the position of the peaks in the reference spectra of pure LiBOB and LiDFOB salt in Fig. S13 and S14.† This deviation in salt oxygen peak positions between experimental and the computational results may possibly be explained by the limits of charge analysis as a tool for PES. The computational results do not take into account factors besides charge (final state effects such as screening) or the computational environment for the analyte is different from the experimental environment, which might affect the kinetic energy of the ejected electrons being analyzed in PES measurements.

In the O 1s spectra, the Li deposition is seen to induce extensive decomposition for both PEO : LiBF4 and PEO:LiBOB, however, minor changes are seen for PEO:LiDFOB (Fig. 8b). A peak that has previously been attributed to Li alkoxide, ROLi, at 529.8 and 530.2 eV for PEO:LiBOB and PEO:LiBF_4_, respectively. The Li alkoxide peak in PEO:LiBF_4_ is wide and could, therefore, consist of several different peaks besides Li alkoxide, however, no other suitable alternatives were found. In the fit, the intensity of the Li alkoxide peak (here at 530.3 eV for PEO:LiDFOB) is diminished to the point of being almost un-observable. The presence of Li alkoxide is corroborated by the simulations with the peak at −1.3|*e*| (Fig. 8d) in all cases. The PES peak at 530.8 eV for PEO:LiBOB and 531.1 eV for PEO:LiDFOB are attributed to the anions coordinating with ionic Li, as shown in the simulations. Based on position, Li oxalate or Li carbonate are candidates for the peak at 529.1 eV in the PEO:LiBOB sample PES spectrum, but as argued in the C 1s section, Li carbonate is not a candidate for this peak since there is no carbonate peak visible in the C 1s spectra. Li oxalate and shifted BOB/DFOB peaks due to coordination between the anion and ionic Li or the Li surface (as suggested by the calculations and mentioned in the C 1s section) are instead stronger candidates for this peak. The shifted oxygen peaks from coordination with Li atoms show up in the charge analysis spectrum at ∼−1.3|*e*|, while the oxygen coordination to the Li surface shows at −1.4|*e*| (Fig. 8d). A new decomposed B–O species appears at −1.6 to −1.7|*e*|, which belongs to oxygen atoms only bonding with boron and coordinating with Li-ions instead of connecting with a carbon atom. Regarding this compound, it, or a similar species, is seen in the PEO:LIBOB PES spectrum at 528.4 eV. In the PEO:LiBF_4_ spectrum at 527.8 eV, a small peak is observed that could be attributed to either this B–O species or to Li oxide. Li oxide has been observed by our group at similar energy before in SPEs that do not contain boron using the same analysis method.^42^ This peak at 527.8 eV in the PEO:LiBF_4_ spectrum is therefore attributed to Li oxide; however this attribution is uncertain due to the low signal-to-noise ratio of the peak.

#### B 1s

For the B 1s PES spectra, the low concentration together with a low cross-section for PE emission resulted in lower quality spectra compared to the other core-levels. The anion peak is found at 194.4 eV for PEO:LiBF_4_, 193.7 eV for PEO:LiBOB, and 194.1 eV for PEO:LiDFOB, while in the charge analysis it is found at +2.4|*e*|, +2.2|*e*|, and +2.3|*e*|, respectively, matching the order in the PES data very well. Another feature can be found at about 187 eV in the PEO:LiDFOB PES spectrum (both before and after deposition), but might be an artefact of noise.

**Fig. 9:**
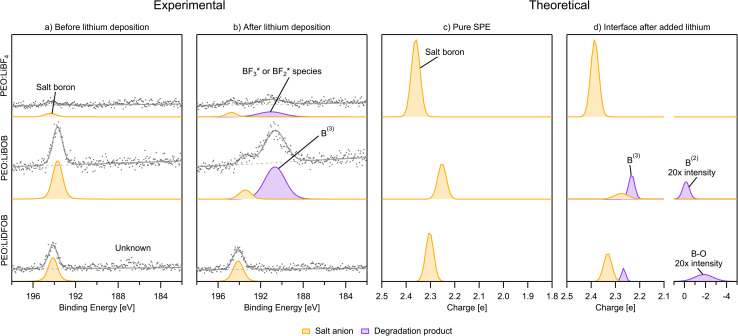
(a) PES B 1s spectra before Li deposition. (b) PES B 1s spectra after deposition. (c) Charge analysis spectra for boron of the pure SPE. (d) Charge analysis spectra for boron at the Li|SPE interface. The intensity of some peaks have been increased in order to improve visibility. PES spectra intensity is not normalized.

After-deposition peaks of decomposition products appear for both PEO:LiBF_4_ and PEO:LiBOB, while no decomposition was experimentally identified for the PEO:LiDFOB. For the PEO:LiBF_4_ SPE, no salt decomposition is found in the AIMD simulation. In order to obtain charge analysis spectra to aid the analysis of the breakdown we see in the PES spectrum, we also investigated the decomposition mechanism by the CI-NEB method (Fig. 3b) and calculated the atomic charge of boron in 
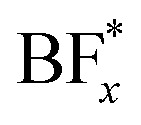
 species. The calculated charge in 
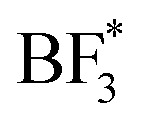
, 
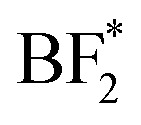
, BF*, and B* are 0.75, −0.58, −2.60, and −4.07|*e*|, respectively. Combined, the simulation and experimental results point towards the likely breakdown products of PEO:LiBF_4_, including 
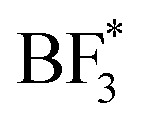
 and 
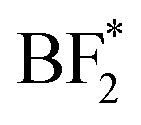
. For PEO:LiBOB, the new peak is interpreted as B^(3)^, also found in the AIMD calculations for PEO:LiBOB (and detailed in Fig. 5). The B^(3)^ species corresponds to the O–B species found in the oxygen spectra, and together, they imply that the oxalate group of the BOB anion has not decomposed to Li oxalate. For PEO:LiDFOB, the only difference in the PES spectra attributable to the deposition is that the main salt peak broadens a bit compared to the one before Li deposition. A low-coordinated boron compound peak emerges at low atomic charge regions (B–O at −2|*e*|) in the charge analysis spectrum for PEO:LiDFOB but is not explicitly seen in the PES spectrum, perhaps due to the low intensity. Thus, we can conclude that the BOB anion degrades more easily than the DFOB anion and that the over-arching trend shows that the PEO:LiDFOB sample degrades the least when exposed to Li. This is corroborated by both the experimental PES (Fig. 6c, 8c, and 9c) and the computational (Fig. 9d) results.

#### F 1s

**Fig. 10:**
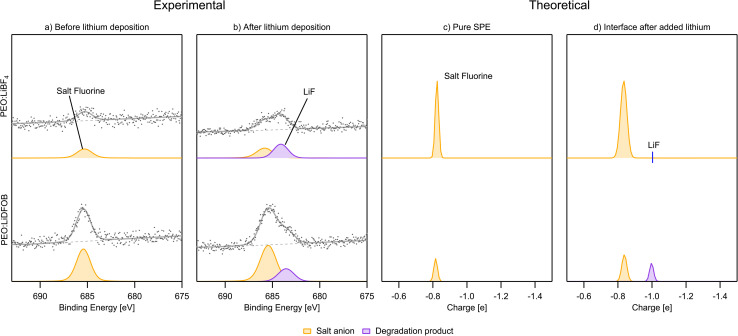
(a) PES F 1s spectra before Li deposition. (b) PES F 1s spectra after deposition. (c) Charge analysis spectra for fluorine of the pure SPE. (d) Charge analysis spectra for fluorine of the Li|SPE interface. PEO:LiBOB is not included since this electrolyte lacks fluorine. PES spectra intensity is not normalized.

For the F 1s spectra, since LiBOB does not contain any fluorine, there are no results for the PEO:LiBOB sample. For PEO:LiBF_4_ and PEO:LiDFOB, before deposition (Fig. 10a), both spectra show only one peak for each sample, corresponding to the salt. This is also seen in the charge analysis spectra. There is less fluorine signal in the PEO:LiBF_4_ F 1s spectrum than in the PEO:LiDFOB spectrum, indicating a lower fluorine concentration on the surface. This is not what is expected from bulk concentrations of the salts. From the bulk concentrations of LiBF_4_ and LiDFOB in the SPEs, the F 1s signal is expected to be larger for the PEO:LiBF_4_ sample than that of the PEO:LiDFOB sample. The fact that the signal is instead smaller means that there is a higher concentration of salt on the surface for the PEO:LiDFOB sample compared to the PEO:LiBF_4_ sample: there's either an accumulation of salt on the surface of PEO:LiDFOB, a depletion of salt on the surface of PEO:LiBF_4_, or both. An accumulation of salt at the surface has been found before, both for liquid electrolytes^69^ and SPEs,^42^ and it is here more clearly demonstrated that the accumulation or depletion is dependent on the chemical composition of the salt. Accumulation or depletion of the salt at the surface of an electrolyte could result in differences in SEI formation; however to what extent this is the case remains to be investigated.

After the deposition of Li, LiF is found in both samples, as expected from previous studies detailed in the introduction. Less breakdown of the salt is again observed for the PEO:LiDFOB sample, as can be seen by comparing the peak areas of the salt F peak and the LiF peak in Fig. 10b. The charge analysis spectra match these results very well for PEO:LiDFOB, showing clear LiF formation as well as matching the intensity of the peak.

### Correlation and discussion of results

The AIMD methodology provided a deep understanding needed to interpret the *in situ* deposition PES spectra with greater accuracy. The generated charge analysis spectra are not a perfect representation of the PES spectra, but in the C 1s, B 1s, and F 1s, a good match is found. Additionally, several experimental conclusions (*e.g.*, the possible polymerization of ethylene or that Li oxalate is not the only decomposition product from the oxalate group) would not be possible without the simulations.

From the combined PES-AIMD results presented in the previous parts, a number of breakdown products from the deposition of Li can be established. From the polymer, Li oxide is seen in small amounts for PEO:LiBF_4_. Another decomposition product is Li alkoxide, which can be seen in all three samples. The relative amounts of Li alkoxide are seen to differ for the different SPEs, in order of increasing amounts from PEO:LiDFOB to PEO:LiBF_4_ to PEO:LiBOB. The PEO:LiBF_4_ and PEO:LiBOB systems also show substantially more hydrocarbons than expected after Li deposition, and as suggested above, this large amount of hydrocarbons indicates polyethylene formation. The AIMD simulations provide final evidence showing the polymerization of ethylene and Li ethylene compounds into polyethylene (Fig. 7) through radical polymerization. However, no polymerization is seen in the simulation of the interface. This can be linked to two main factors: concentration and steric hindrances. First, the simulation of the interfaces starts with no ethylene and has at most 6 ethylene. The simulation of the polymerization reaction has 12 ethylene from the start, and the simulation box of the polymerization simulation is approximately 1/4 of the volume of the interface simulation. This means that the concentration of ethylene is much lower in the simulation of the interface compared to the simulation of the polymerization. Second, the polymerization simulation only contains ethylene and Li, while the interface simulation contains other compounds, making the likelihood of contact between ethylene molecules lower. There are steric hindrances to the polymerization in the simulation of the interface. Taken together, this means that the frequency of polymerization reactions can be expected to be orders of magnitude lower in a simulation of the interface compared to what it is in the simulation of the polymerization reaction, and it is unlikely that we could observe it in a simulation of the interface within a reasonable timeframe. This explains why polymerization was observed in the polymerization simulation (Fig. 7) but not in the interface simulation (Fig. 3 and 4). The time frame of the PES experiment is on a much longer time scale than either simulation. It would allow for the full reaction to take place, which makes polyethylene a likely explanation for the large hydrocarbon peak. The extent and implication of polyethylene formation in a battery is still to be determined. It is uncertain if polyethylene plays a role in observed poor long-term stability; it could also be the case that more polyethylene is observed as more decomposition takes place, and the amount of systems investigated is also limited, but it is noted that the two SPEs with polyethylene as a breakdown product from Li deposition (PEO:LiBF_4_ and PEO:LiBOB) show worse long-term stability than the one without polyethylene (PEO:LiDFOB). Regardless, the effects of polyethylene in the SEI is an interesting topic that requires further investigation.

For the salts, several different decomposition products can be observed. For PEO:LiBF_4_, LiF, and the 
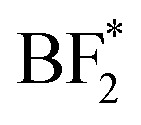
 or 
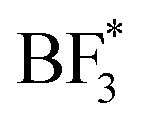
 species seem to be the majority of the salt decomposition products. When it comes to LiBOB, there exist many possibilities for the breakdown of products. Coordination between the BOB anion and Li, different oxalates, borates with different coordination numbers of the boron, and Li oxalate are likely products. Regarding the Li oxalate, it should be noted that it is not likely to be the only end-product of the decomposition of the oxalate group of the BOB anion, as the AIMD simulations clearly show the breaking of the O–C bond in the oxalate group of the BOB anion. This holds true even if the extent of decomposition is lesser in the AIMD simulations, as the O–C bond would have to reform for Li oxalate to form. For PEO:LiDFOB, only a few decomposition compounds can be seen. LiF is the most prominent of these, but also Li oxalate or the anion coordinating strongly with Li, and possibly some B^(3)^ species.

From the observations in the PES and AIMD results, it is clear that PEO:LiDFOB decomposes the least during the deposition of Li, which could be a reason for the comparatively high capacity retention seen in the galvanostatic cycling results. With the assumption that the PES measurements at room temperature and the simulations at 400 K are representative of the cycling data at 60 °C, these data suggest that the SEI created from the PEO:LiDFOB SPE is stable even though it requires less decomposition than the other SPEs in this study. Regarding the fluorinated part of LiDFOB, a study on “model LiF-enriched SEIs” found that the usefulness of fluorinated salts is rapid SEI formation, a result of them reacting early during the charging process (at higher potentials).^70^ Regarding the oxalate and borate in LiDFOB, one of the first studies done on LiBOB shows that the presence of LiBOB in a propylene carbonate-based electrolyte can create an SEI which is mechanically stable, as the addition of LiBOB in the electrolyte prevented the graphite exfoliation commonly seen for these liquid systems.^32^ This poses the possibility that the oxalate and borate decomposition products could help the mechanical stability of the rapidly formed LiF-based decomposition layer. Another study on SEI formation from LiDFOB compared to LiBOB + LiBF_4_ as the salt in a liquid electrolyte system found that LiDFOB forms a more uniform LiF-based SEI film than LiBOB + LiBF_4_. This leads to more uniform Li deposition for the LiDFOB system, according to the authors.^71^ If any of these effects are present in the PEO:LiDFOB sample is hard to conclude due to PEO being a solid, which limits the movement of large anions and decomposition products. Regardless, the SEI created from LiDFOB (half LiBOB, half LiBF_4_) seems to require very little breakdown to be effective. On the other hand, LiDFOB is also known to increase cycling performance in cells with high-voltage cathodes,^72^ but stability at the cathode should not play a significant role here since the cells were cycled with LFP using a cutoff voltage of 4.0 V *vs.* Li^+^/Li. This is further corroborated by our previous study on similar SPEs,^73^ in which similar trends in coulombic efficiency was observed regardless of cell type (Cu *vs.* LFP full cells or Cu *vs.* Li half cells).

## Conclusion

By combining *in situ* deposition PES, AIMD simulations, and galvanostatic cycling in order to investigate three SPE materials (PEO:LiBOB, PEO:LiBF_4_, and PEO:LiDFOB), insight into the anode–electrolyte interface and its connection to the long-term stability of SPE-based batteries is gained.

The main conclusion from this study is that the PEO-based polymer electrolyte containing LiDFOB displays several, but not all, decomposition products of the SPEs containing LiBOB and LiBF_4_, and with a lower degree of salt and polymer breakdown than the other SPEs. Although generating less decomposition products, the formed interfacial layer in PEO:LiDFOB still seems to form an effective SEI, as seen in the relatively high capacity retention compared to the other SPEs. As there are similar products between these employed salts, the better performance of the PEO:LiDFOB system cannot be related to any specific breakdown products. Instead, it is discussed that the combination of LiF, together with boron and oxalate breakdown products, provide higher mechanical stability and uniformity of the SEI.

Furthermore, the SEI formed by SPEs based on PEO could contain polyethylene as a major decomposition component. A reaction mechanism obtained in AIMD simulation shows that the breakdown of PEO can form ethylene and Li ethylene, which in turn polymerize to polyethylene. This conclusion is also supported by the experimental PES results showing the formation of large amounts of hydrocarbons. Assuming this is the case also in the battery cell, the presence of polyethylene, in turn, is accompanied by worse cycling performance. The effects of polyethylene as part of the SEI require additional investigation.

## Conflicts of interest

There are no conflicts to declare.

## Supplementary Material

TA-012-D3TA07175H-s001
