# Supplementary material for: Initial SEI formation in LiBOB-, LiDFOB- and LiBF4-containing PEO electrolytes
Source: J Mater Chem A Mater. 2024 Mar 19;12(15):9184–99. doi: 10.1039/d3ta07175h (PMC11019830; doi:10.1039/d3ta07175h)
Supplement: TA-012-D3TA07175H-s001 [file TA-012-D3TA07175H-s001.pdf]

Electronic Supplementary Material (ESI) for Journal of Materials Chemistry A.
This journal is © The Royal Society of Chemistry 2024

Tables and figures for Supplementary Information

Table S1. Compositions of four electrolyte mixture systems in the AIMD calculations.

Systems	# of PEO	# of Li Salt	wt%	Density (g/mL)
**PEO:LiDFOB**	8	5	23.4	1.19
**PEO:LiBOB**	8	4	24.8	1.21
**PEO:$LiBF_4$**	8	8	24.2	1.20

Table S2. Calculated binding energy of Li-ion on PEO polymer chain and salt anions

Systems	Binding type	Binding energy (eV)
**PEO**	Coiled	-3.00
	Anchored	-0.86
**BF_4^-**	F…Li…F	-1.86
**BOB**	$O_{carbonyl}$…Li…$O_{carbonyl}$	-1.68
	O_{alkoxy}…Li…O_{alkoxy}	-1.25
	$O_{carbonyl}$…Li…O_{alkoxy}	-1.30
**DFOB**	$O_{carbonyl}$…Li…$O_{carbonyl}$	-1.89
	$O_{carbonyl}$…Li…O_{alkoxy}	-1.49
	O_{alkoxy}…Li…F	-1.53
	F…Li…F	-1.58

Table S3. Catalog of electrolytes decomposition reactions and the approximate time they occurred during AIMD simulations.

Systems	Timepoint (fs)	Reaction	
**PEO:LiBF$_4$**	5070, 5230, 6925, 10300, 11060, 11105, 12520	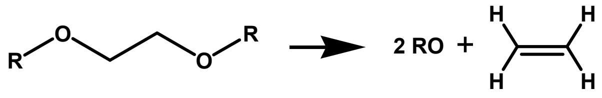	PEO decomposition
**PEO:LiBOB**	1160	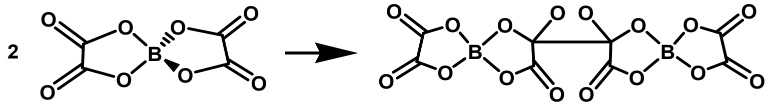	Salt dimerization
	2920, 8060	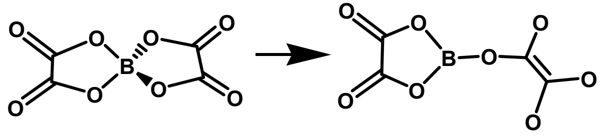	Salt ring-opening

	5090	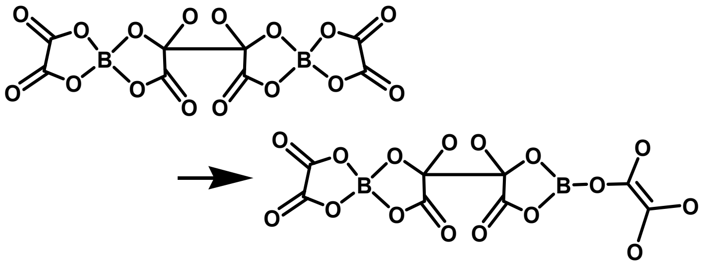	Salt ring-opening
PEO:LiBOB	7140	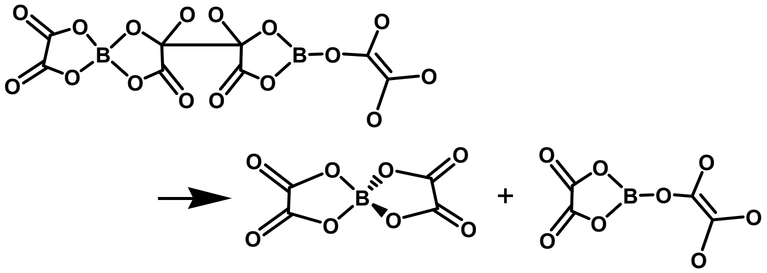	Salt dimer decomposition
	8230	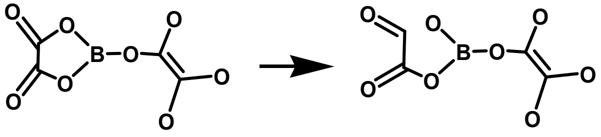	Salt ring-opening

	8730	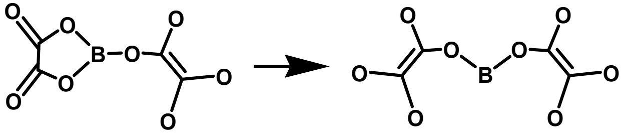	Salt ring-opening
	11030	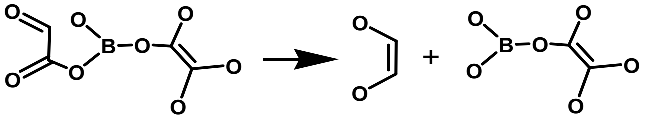	Salt decomposition
**PEO:LiBOB**	8100, 11030	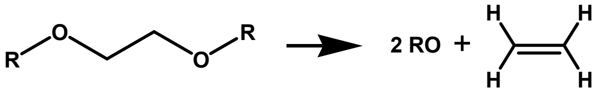	PEO decomposition
**PEO:LiDFOB**	1740, 11630	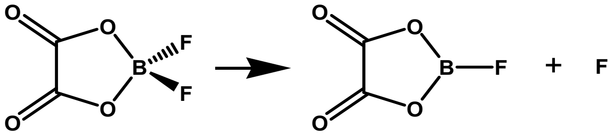	Salt decomposition
	2120	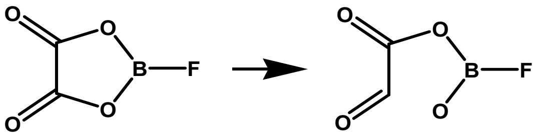	Salt ring opening

	11060	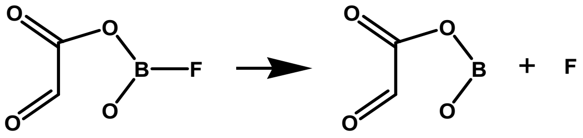	Salt decomposition
	11960	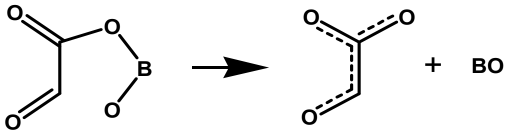	Salt decomposition
**PEO:LiDFOB**	5250, 7200, 8030, 8240, 11100, 11120	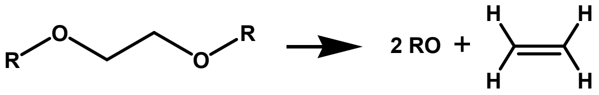	PEO decomposition

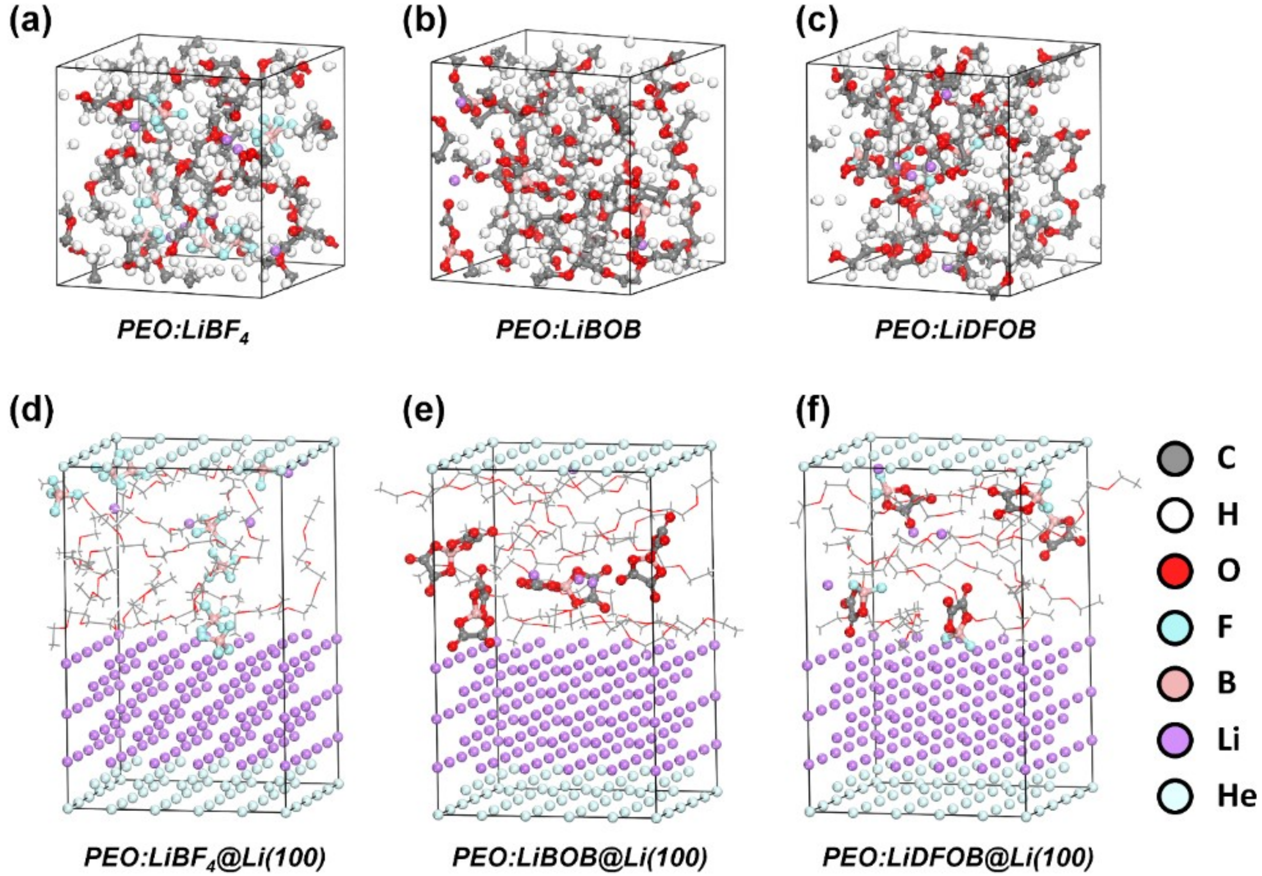

Figure S1. AIMD simulation box for (a) PEO:$LiBF_4$ (b) PEO:LiBOB, (c) PEO:LiDFOB electrolyte, and (d) PEO:$LiBF_4$ (e) PEO:LiBOB (f) PEO:LiDFOB electrolyte on the Li (100) surface.

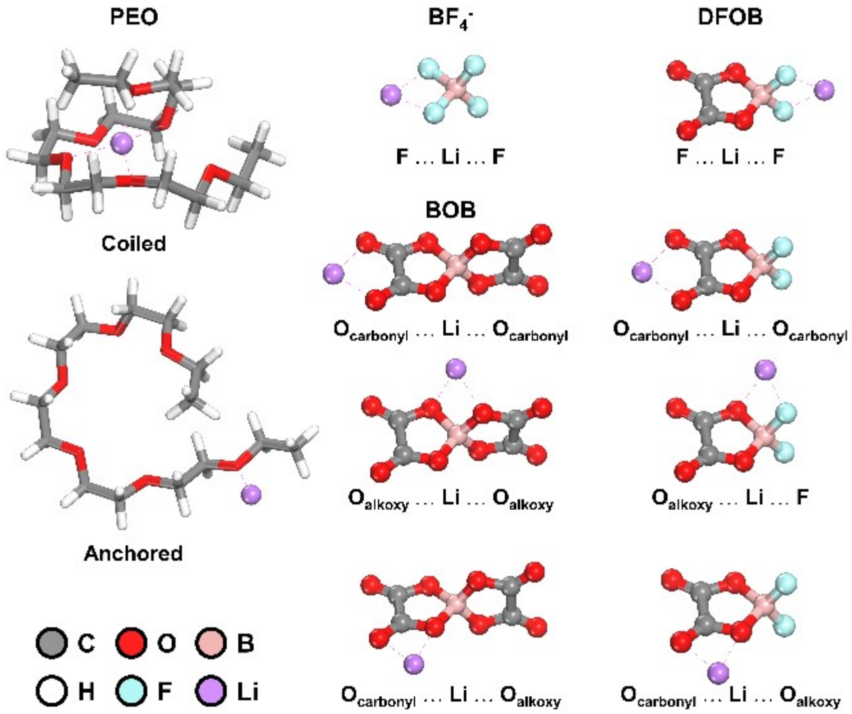

Figure S2. The optimized structures of Li^+ binding with PEO chain, BF_4^-, BOB, and DFOB anions by different binding types

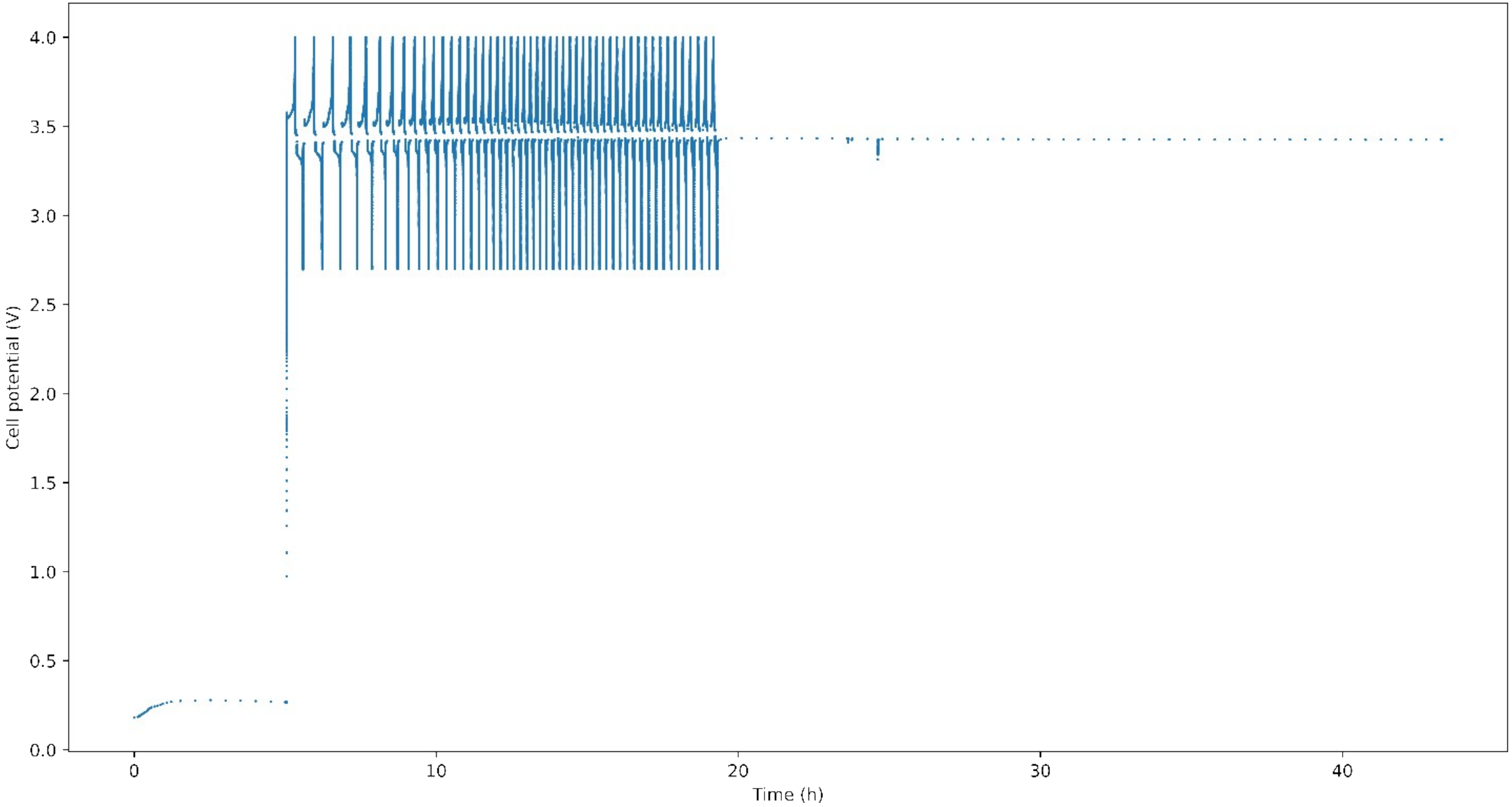

Figure S3. Cycling curves for PEO:$LiBF_4$ Cu vs. LFP cell.

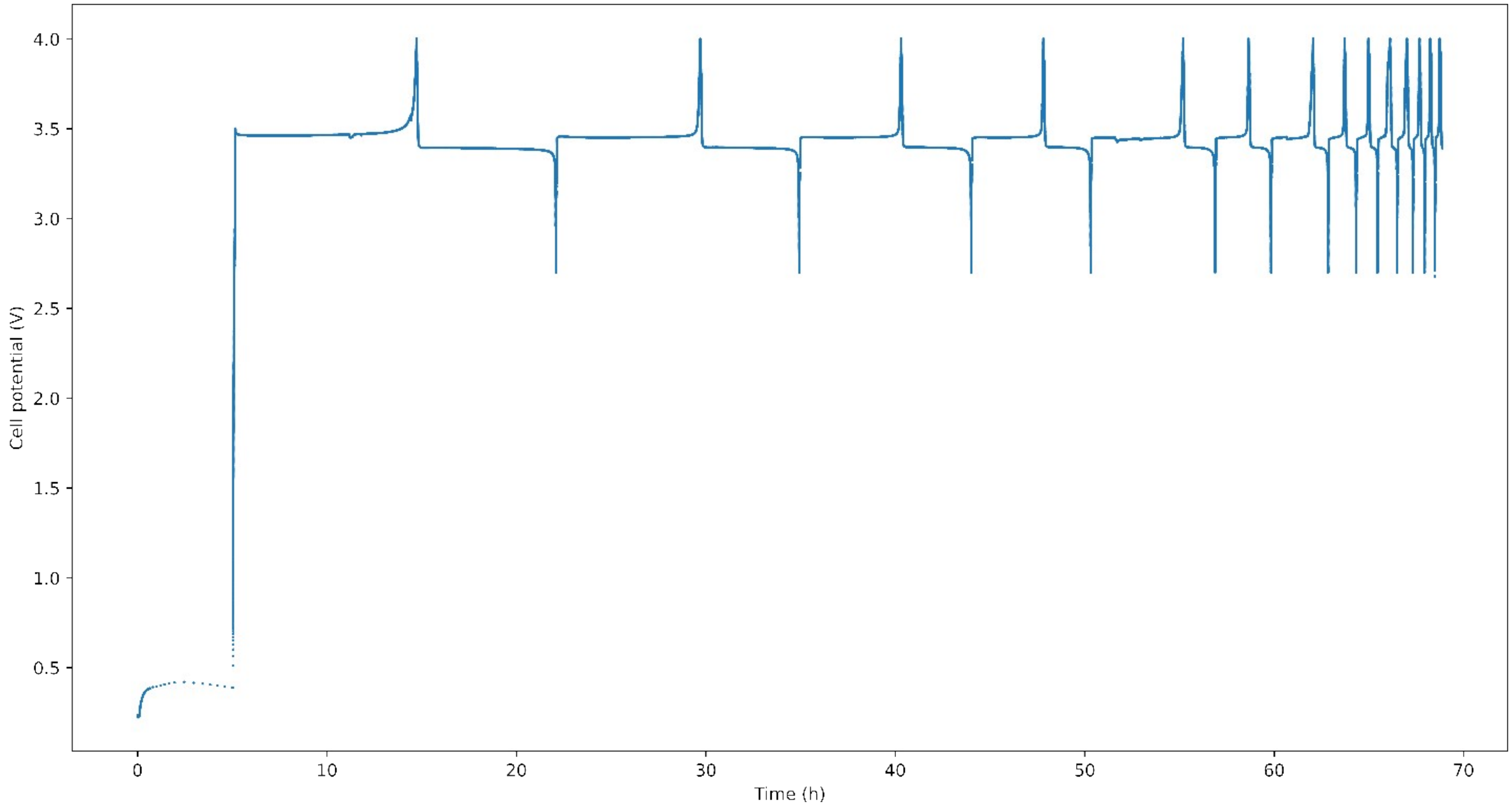

Figure S4. Cycling curves for PEO:LiBOB Cu vs. LFP cell.

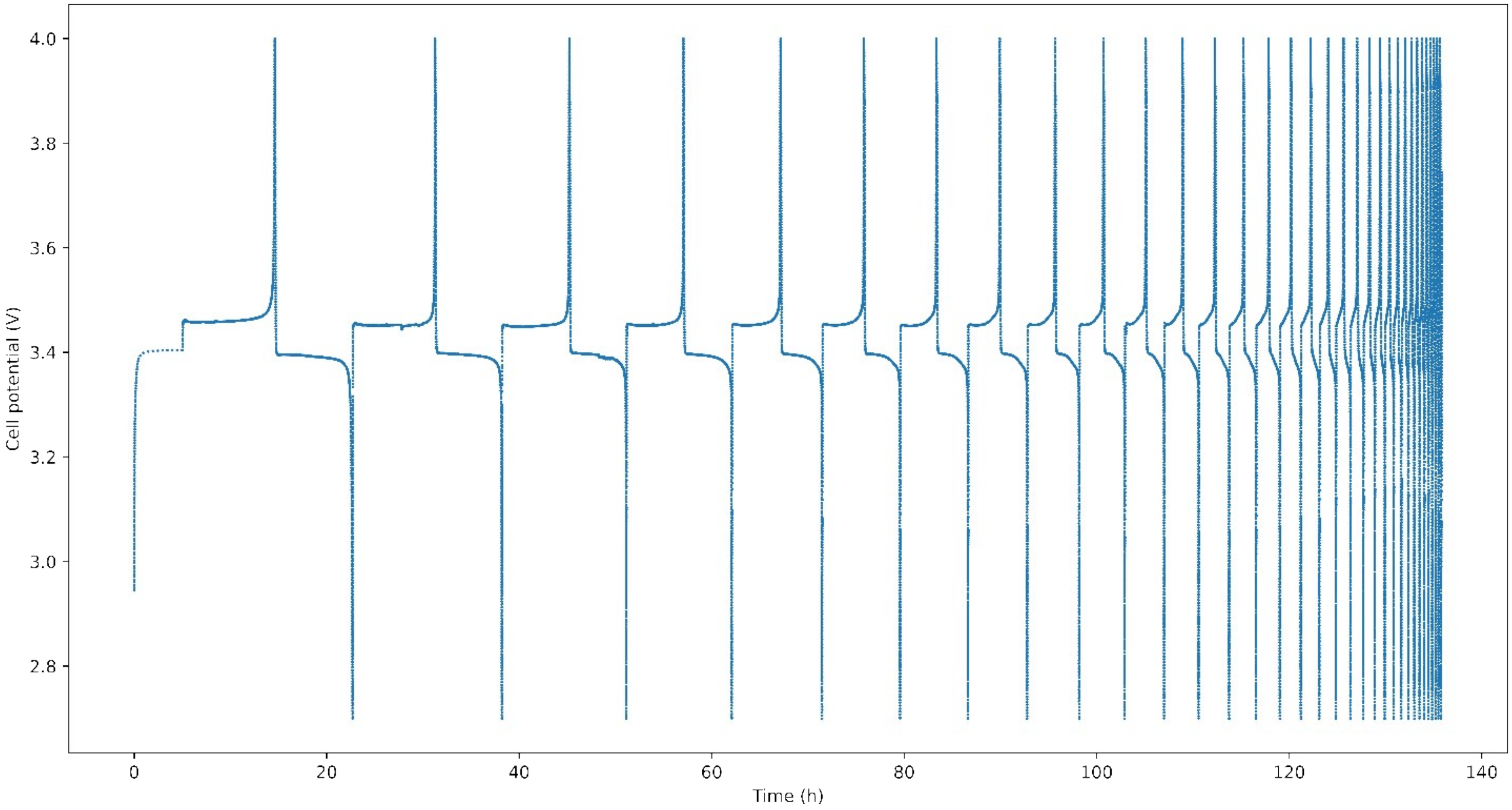

Figure S5. Cycling curves for PEO:LiDFOB Cu vs. LFP cell.

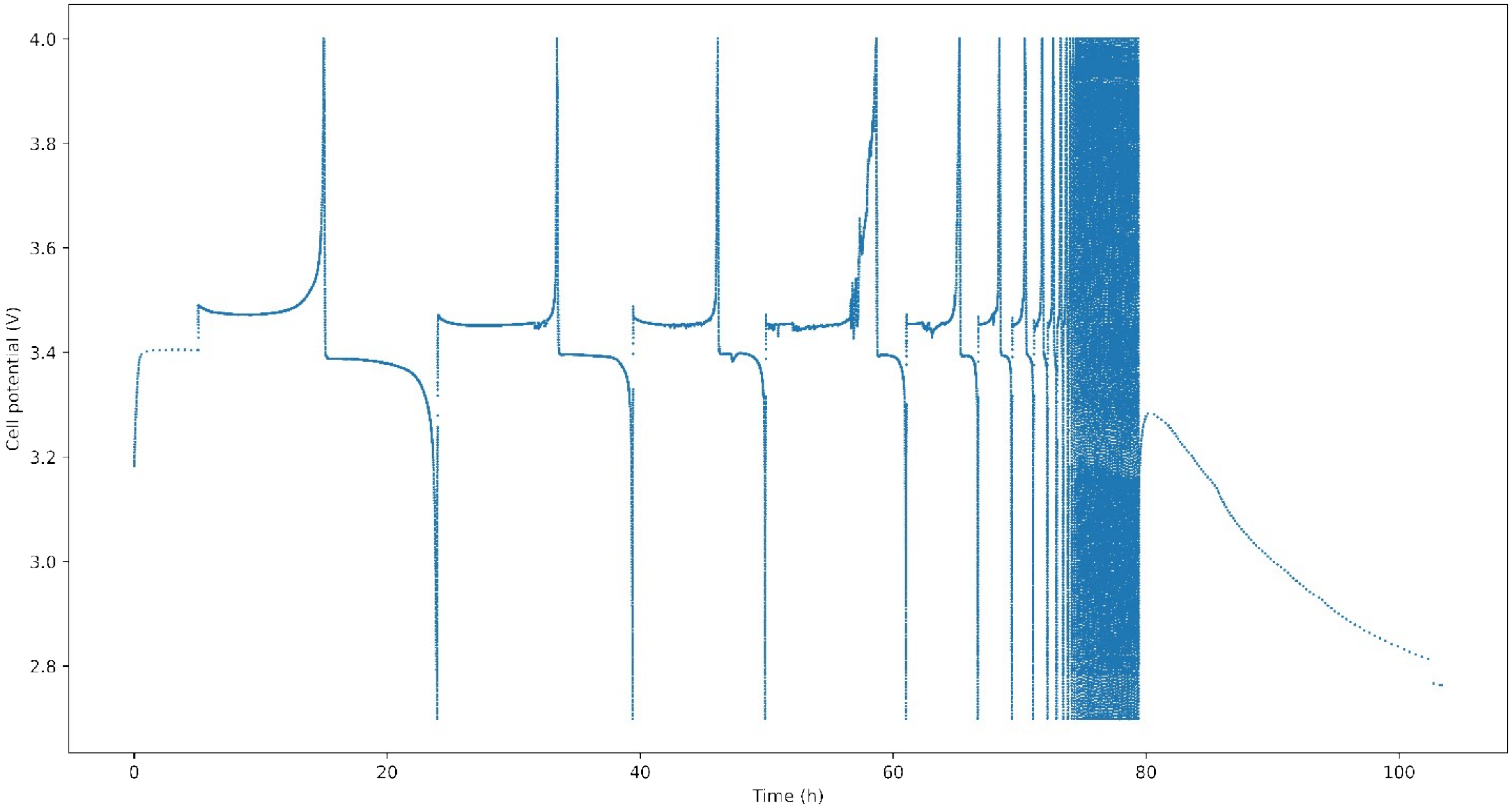

Figure S6. Cycling curves for PEO:LiTFSI Cu vs. LFP cell.

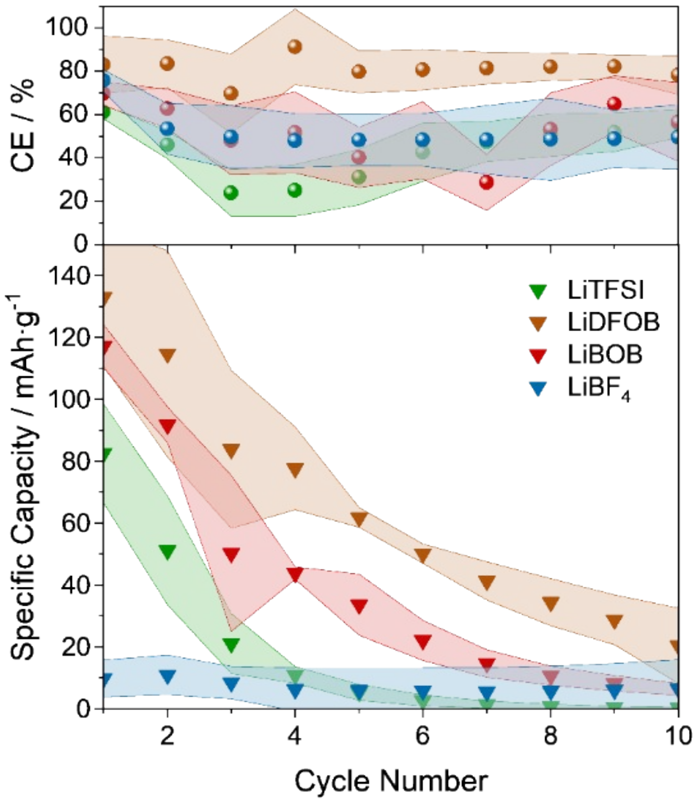

Figure S7. Coulombic efficiency and specific capacity of anode-less Cu vs. LFP full cells using either PEO:LiTFSI, PEO:$LiBF_4$, PEO:LiBOB or PEOLiDFOB as the electrolyte.

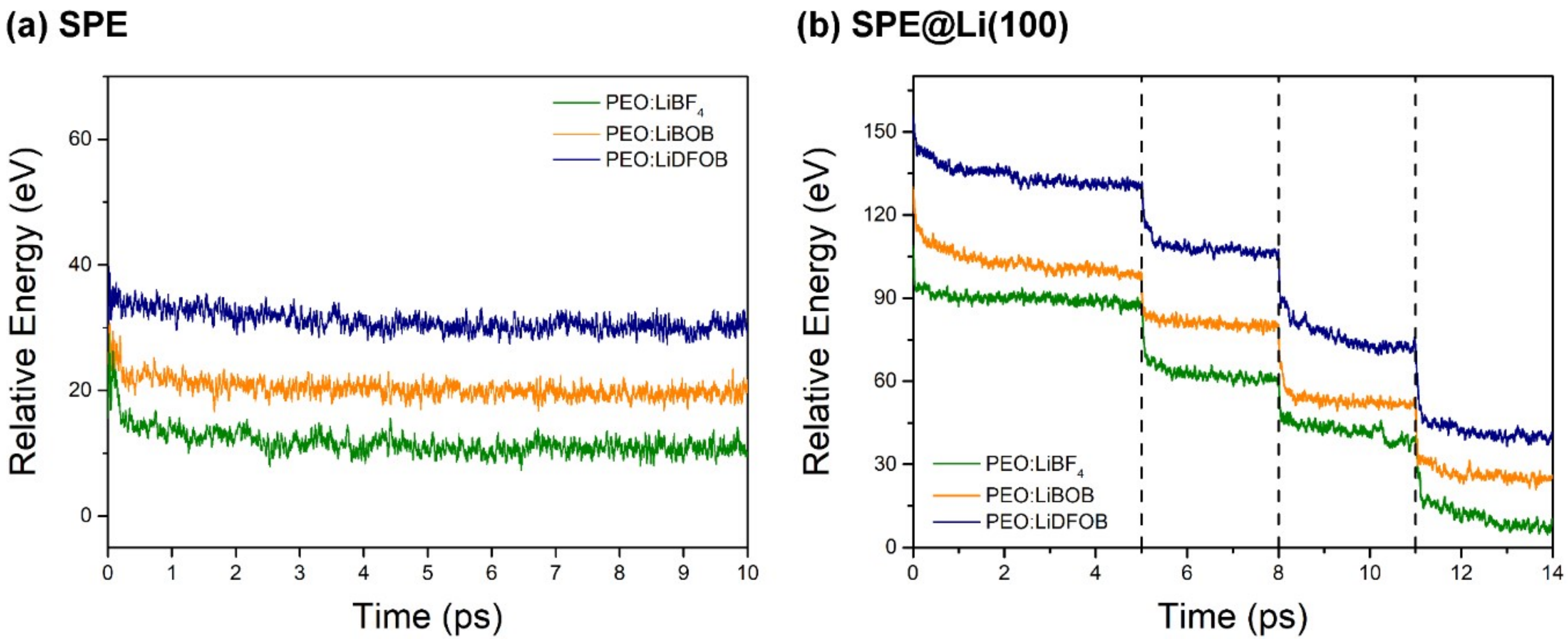

Figure S8. Energy fluctuations over the simulation time for (a) PEO:$LiBF_4$, PEO:LiBOB, and PEO:LiDFOB electrolyte, and (b) the three SPEs on a Li (100) anode surface.

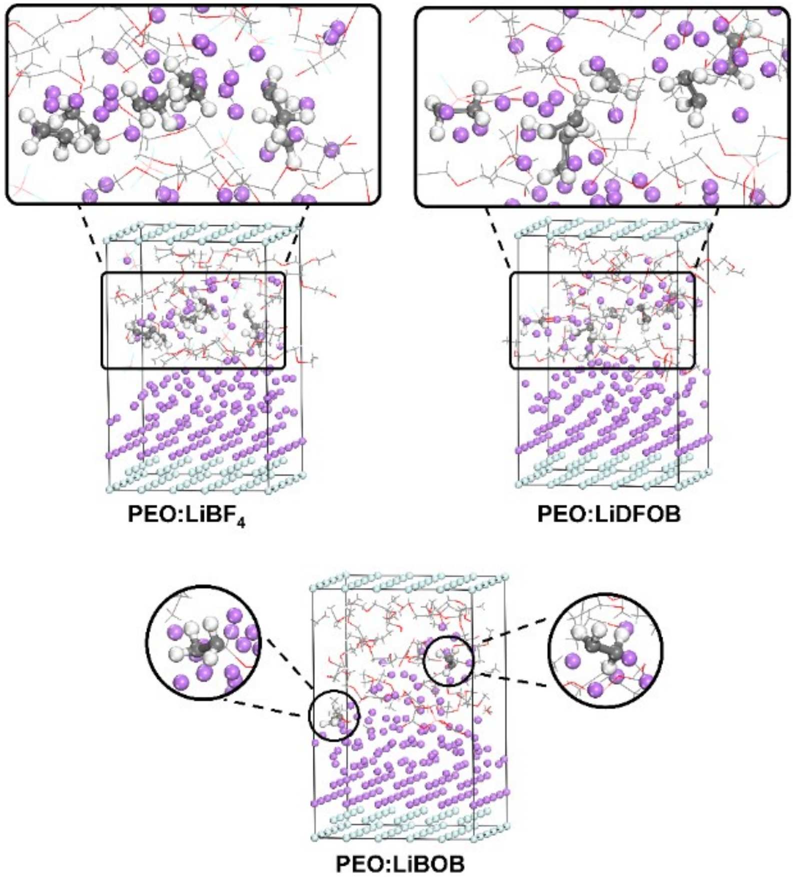

Figure S9. Final state of the three SPE systems at the Li interface, showing the formed Li ethylene complexes.

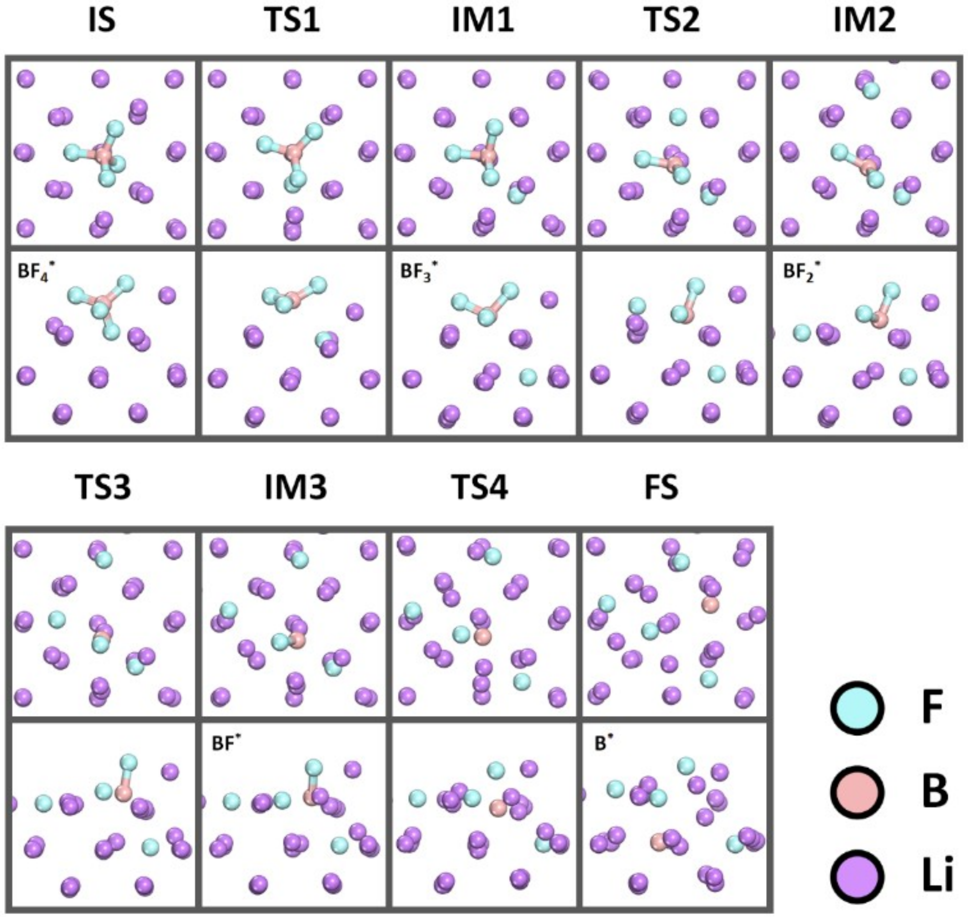

Figure S10. Top and side views of initial state (IS), intermediates (IM), transition states (TS), and final state (FS) for BF_4^* decomposition on the Li (100) surface.

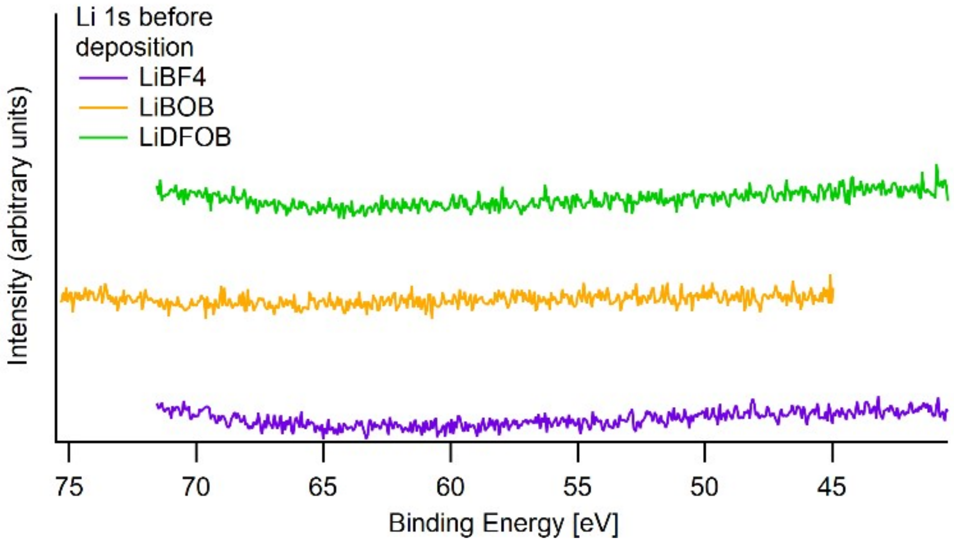

Figure S11. Li 1s spectra for PEO:$LiBF_4$, PEO:LiBOB and PEO:LiDFOB before deposition.

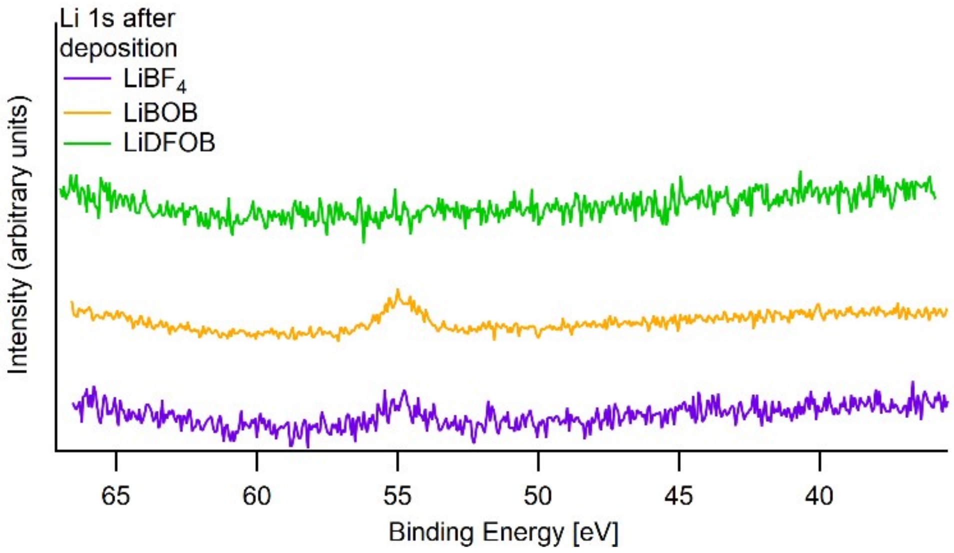

Figure S12. Li 1s spectra for PEO:$LiBF_4$, PEO:LiBOB and PEO:LiDFOB after deposition.

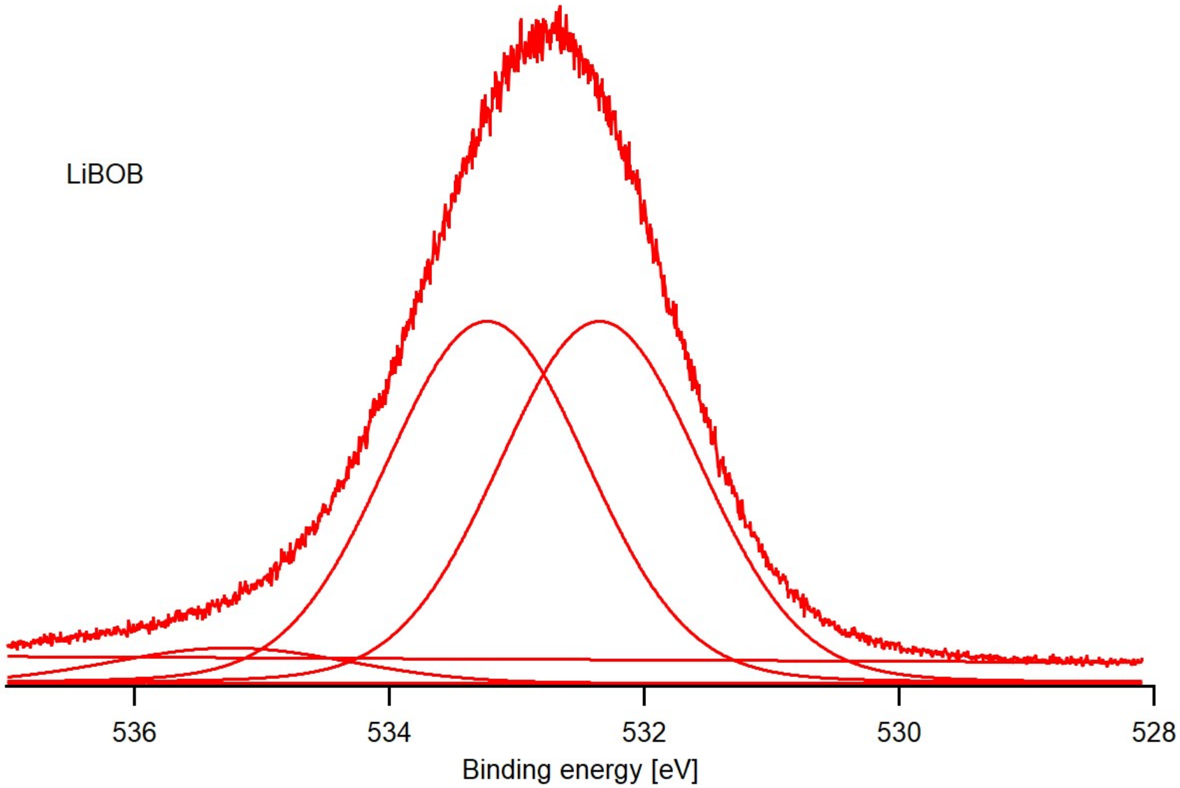

Figure S13. Reference spectrum of LiBOB salt.

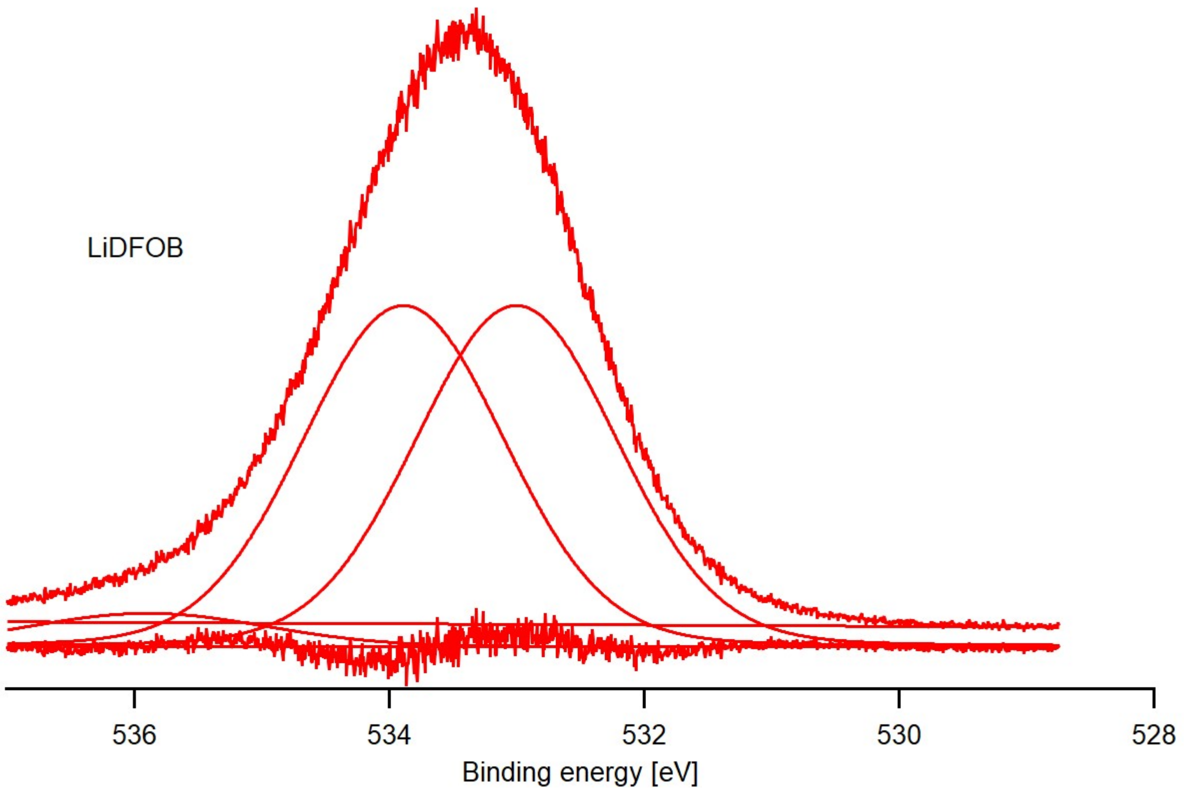

Figure S14. Reference spectrum of LiDFOB salt.
